# Proteomic blood-based biomarkers of brain damage in traumatic brain injury: are they suitable surrogate endpoints for cerebral pressure autoregulatory-guided therapy?

**DOI:** 10.1186/s13054-026-06132-4

**Published:** 2026-06-13

**Authors:** Rozerin Kevci, Anders Hånell, Marcel Aries, Cecilia Åkerlund, Andras Buki, Shubhayu Bhattacharyay, Guido Di Tommaso, Rik Hendrix, Virginia Newcombe, Anders Lewén, Per Enblad, Erta Beqiri, Peter Smielewski, Teodor Svedung Wettervik, the HR subgroup CENTER-TBI collaborators

**Affiliations:** 1 Department of Medical Sciences, Section of Neurosurgery, Uppsala University, Uppsala, Sweden; 2 Department of Intensive Care, Maastricht University Medical Center+, Maastricht, The Netherlands; 3 Mental Health and Neurosciences Institute, University Maastricht, Maastricht, The Netherlands; 4 Department of Physiology and Pharmacology, Section for Perioperative Medicine and Intensive Care, Karolinska Institute, Stockholm, Sweden; 5 Function Perioperative Medicine and Intensive Care, Karolinska University Hospital, Stockholm, Sweden; 6 Faculty of Medicine and Health, School of Medical Sciences, Örebro University, Örebro, Sweden; 7 Harvard Medical School, Boston, USA; 8 Department of Neurointensive Care Unit, ASST Grande Ospedale Metropolitano Niguarda, Milan, Italy; 9 Department of Medicine, University of Cambridge, Cambridge, UK; 10 Brain Physics Laboratory, Division of Neurosurgery, Department of Clinical Neurosciences, University of Cambridge, Cambridge, UK

**Keywords:** Biomarkers, Cerebral autoregulation, Cerebral pressure autoregulation, Intracranial pressure, Intensive care, Pressure reactivity index, Traumatic brain injury

## Abstract

**Background:**

Management after severe traumatic brain injury (TBI) aims to prevent secondary injury by optimizing cerebral physiology, yet conventional metrics such as intracranial pressure (ICP) and cerebral perfusion pressure (CPP) incompletely capture the underlying pathophysiology. Impaired cerebral pressure autoregulation (CPA) is common and may exacerbate secondary injury. Proteomic blood-based biomarkers (PBBMs) of astrocytic (glial fibrillary acidic protein [GFAP], S100 calcium-binding protein B [S100B]), and neuronal/axonal injury (total tubulin associated unit [t-Tau], neurofilament light chain [NfL], ubiquitin C-terminal hydrolase-L1 [UCH-L1], neuron-specific enolase [NSE]) may provide global indicators of secondary injury. This study investigated temporal associations between PBBMs and cerebral physiological variables (ICP, CPP, pressure reactivity index [PRx], and CPP deviation from “optimal” CPP [ΔCPPopt]), and evaluated the PBBMs as surrogate short-term endpoints.

**Methods:**

This retrospective, observational multi-center study, used prospective data from the CENTER-TBI cohort and included 151 patients with high-frequency cerebral physiological data and serial PBBM measurements during the first seven days post-injury. Associations were analyzed using Spearman correlations, univariate and multivariate linear mixed effects models (LMEMs), as well as cross correlation analyses adjusted for repeated measures and clinical confounders.

**Results:**

Elevated PBBM levels on day 1 were associated with a greater cumulative burden of high ICP, impaired CPA (positive PRx), and negative ΔCPPopt over the subsequent seven days. Throughout the monitoring period, higher median PBBM concentrations correlated with elevated ICP and impaired CPA, with strongest associations when cerebral physiological monitoring and PBBMs were analyzed from the same day. In multivariate LMEMs, elevated ICP and most PBBMs remained significantly associated, while particularly PRx amplified this association.

**Conclusions:**

Cerebral physiological disturbances during intensive care after TBI were associated with elevated PBBM levels, with ICP showing the strongest influence and impaired CPA amplifying some PBBM responses. Associations were strongest in same-day analyses and appeared bidirectional. These findings suggest that PBBM may reflect the burden of secondary cerebral insults and may serve as candidate endpoints for future trials targeting cerebral physiology, pending validation in prospective high-resolution studies. Importantly, our results highlight the potential for PBBMs to complement multimodal neuromonitoring by tracking secondary injury trajectories.

**Supplementary Information:**

The online version contains supplementary material available at 10.1186/s13054-026-06132-4.

## Introduction

Management following moderate-to-severe traumatic brain injury (TBI) aims to improve functional outcomes by minimizing secondary insults through neuromonitoring and timely interventions. A central focus in this approach is to prevent and treat elevated intracranial pressure (ICP) and to maintain a balanced cerebral perfusion pressure (CPP) to avoid ischemia and hyperemia [1–3]. However, it is recognized that these cerebral physiological measures do not fully capture or reflect the pathophysiological processes leading to neuronal injury after TBI [4]. For instance, many TBI patients lose the ability to maintain cerebral pressure autoregulation (CPA), meaning that ischemia or hyperemia can occur despite “normally accepted” CPP values [5, 6]. To address this and guide treatment, derived CPA indices such as the pressure reactivity index (PRx) and its derived “optimal” CPP (CPPopt) have been developed to quantify CPA and individualize CPP management [5–7]. A recent phase II trial showed that CPPopt-guided therapy is both feasible and safe, paving the way for future effectiveness and outcome trials [8]. However, long-term functional outcome is a rather coarse and multifaceted measure that is influenced by a plethora of factors such as demography, complex injury patterns, clinical course, care trajectories, and neurorehabilitation. Thus, it is a challenge to prove improved functional outcome by changing only one of these many variables. Consequently, there is a need for surrogate endpoints to evaluate any potential physiological and treatment effect for upcoming CPA-guided management studies.

An alternative approach has been to assess downstream physiological monitoring variables such as invasive brain tissue oxygenation or cerebral energy metabolism via microdialysis [4, 9, 10]. However, these modalities provide highly focal measurements that may not accurately reflect global cerebral pathophysiology – particularly in the context of TBI, which often presents with a spatially heterogenous injury pattern [4]. In contrast, proteomic blood-based biomarkers (PBBMs) of central nervous system (CNS) injury represent global indicators of brain injury and may be more suitable endpoints for secondary brain injury. Most extensively studied PBBMs include glial fibrillary acidic protein (GFAP), neurofilament light chain (NfL), neuron-specific enolase (NSE), S100 calcium-binding protein B (S100B), total tubulin associated unit (t-Tau), and ubiquitin C-terminal hydrolase-L1 (UCH-L1) [10, 11]. These PBBMs reflect different kinds of cerebral damage including astrocytic damage, blood-brain barrier compromise [12, 13], neuronal cell body necrosis [14], and axonal disruption [13]. Importantly, larger studies have demonstrated that several of these PBBMs are independently associated with long-term outcomes in the entire spectrum of TBI severity [10–12, 15–21]. A recent novel framework for TBI-classification incorporates PBBMs as a key pillar to stratify the severity of the primary disease [22, 23]. Because PBBMs differ in cellular origin, specificity, and clearance, the clinical interpretation must consider these biological distinctions. In previous studies, GFAP and UCH-L1 have shown stronger associations with dynamic cerebral physiology (elevated ICP, reduced CPP, and impaired CPA) than traditional PBBMs like NSE or S100B, whose extracranial expression reduces specificity and may weaken such associations in the acute phase [12, 14–16]. Notably, NfL is released slowly from damaged axons and has a prolonged serum half-life of days to weeks [13, 23–25], resulting in a gradual accumulation pattern that is distinct from cytoplastic proteins such as GFAP and UCH-L1, which peak more rapidly after acute injury. This kinetic heterogeneity has direct implications for the temporal associations examined in the present study.

The relationship between cerebral physiological disturbances and PBBM release is likely bidirectional, is likely bidirectional, though the direction and magnitude of these associations cannot be established from observational data alone. Severe primary brain injury, often accompanied by an early surge in PBBMs, which reflects the overall burden of injury rather than type, may be followed by a more adverse trajectory of cerebral pathophysiology [26]. Conversely, pronounced secondary insults, such as impaired CPA and reduced CPP, can exacerbate neuronal damage and drive further PBBM release. Thus, PBBMs may not indicate primary injury severity and disease trajectories, but also capture the cumulative impact of secondary injury, offering potential as candidate endpoints for treatment effects on cerebral physiology. Beyond their potential as trial endpoints, PBBMs may add clinically meaningful information in real-time intensive care unit (ICU), complementing cerebral physiological monitoring by capturing biochemical impact of cerebral physiological disturbances.

Using prospectively collected data from the multi-center Collaborative European NeuroTrauma Effectiveness Research in TBI (CENTER-TBI) cohort, we conducted a retrospective analysis to investigate the relationship between PBBMs of brain injury and key cerebral physiological variables. Our objective was to evaluate whether these PBBMs could serve as feasible surrogate endpoints in future trials on CPA-guided therapy in TBI. We also sought to explore the temporal dynamics and resolution of these associations, to inform optimal sampling strategies and PBBM selection.

## Materials and methods

### Material

The study used prospective data from the retrospective, observational study within the CENTER-TBI cohort, which enrolled 277 patients with high-frequency data between 2014 and 2017 from 21 recruiting centers. Of these 277 patients, the final cohort comprised 151 TBI cases with at least 12 h of ICP monitoring and available PBBM sample during the first seven days post-injury (Supplemental Fig. 1).

The CENTER-TBI study (EC grant 602150) has been conducted in accordance with all relevant laws of the EU if directly applicable or of direct effect and all relevant laws of the country where the recruiting sites were located, including but not limited to, the relevant privacy and data protection laws and regulations (the “Privacy Law”), the relevant laws and regulations on the use of human materials, and all relevant guidance relating to clinical studies from time to time in force including, but not limited to, the ICH Harmonised Tripartite Guideline for Good Clinical Practice (CPMP/ICH/135/95) (“ICH GCP”) and the World Medical Association Declaration of Helsinki entitled “Ethical Principles for Medical Research Involving Human Subjects”. Informed Consent by the patients and/or the legal representative/next of kin was obtained, accordingly to the local legislations, for all patients recruited in the Core Dataset of CENTER-TBI and documented in the e-CRF.

#### Ethical approval

was obtained for each recruiting site. The list of sites, Ethical Committees, approval numbers and approval dates can be found on the website: https://www.center-tbi.eu/project/ethical-approval.

### Clinical data management

Data for the CENTER-TBI study has been collected through the Quesgen e-CRF (Quesgen Systems Inc, USA), hosted on the INCF platform and extracted via the INCF Neurobot tool (INCF, Sweden). For patient monitoring and data collection in the High-Resolution repository, the ICM+ research platform (Cambridge Enterprise Ltd, University of Cambridge, UK) and/or Moberg Neuromonitoring system (Moberg Research Inc., USA) were used.

Demographic and low-resolution data were accessed via Opal software [4.6.28] on the 17th March 2024, included age, injury mechanism, admission status (Glasgow Coma Scale [GCS] and pupillary status), Marshall computed tomography (CT) score, imaging data (intracranial bleeding types and volume), Injury Severity Score (ISS), and six-month Glasgow Outcome Scale Extended (GOSE).

### Proteomic blood-based biomarker data

Blood sampling for determination of PBBMs GFAP, NfL, NSE, S100B, t-Tau, and UCH-L1 has been described in detail elsewhere [27]. In short, blood samples were obtained within 24 h of injury and generally once a day thereafter using gel-separator tubes. After 45 ± 15 min of coagulation in room-temperature, blood was centrifuged and aliquoted (8 × 0.5 mL) within 60 min of collection. Later on, the samples were frozen at -80℃ before shipment to the CENTER-TBI biobank (Pécs, Hungary) for biochemical analysis [23]. The Human Neurology 4-Plex B assay, based on Single-Molecule Arrays (SiMoA), was utilized to measure GFAP, NfL, t-Tau, and UCH-L1 using the SR-X benchtop platform (Quanterix Corp., Lexington, MA) [28, 29]. Meanwhile, NSE and S100B levels were assessed on the e602 module of the Cobas 8000 modular analyzer (Roche Diagnostics, Mannheim, Germany) with electrochemiluminescence immunoassay kits (Elecsys S100 and NSE assays) [30].

### Cerebral physiological data

Either an intraparenchymal strain gauge probe (Codman ICP MicroSensor: Codman & Shurtleff Inc., Raynham, MA, USA) or a fiber optic pressure sensor (Camino ICP Monitor, Integra Life Sciences, Plainsboro, NJ, USA) was used as ICP monitors. The decision to initiate invasive ICP monitoring was generally made in accordance with the international TBI guidelines [31], though local management protocols may have led to some variations in practice. This applies to the general TBI patient management protocol as well.

High-frequency (100 Hz or higher) physiological data acquisition and processing, including artifact removal and down-sampling into 10-second averaged time series of the cohort, have been described in detail in previous work [32]. The ICM+ research software platform was employed for data processing [33]. In summary, 10-second averaged arterial blood pressure (ABP) and ICP time series were analyzed. The PRx was calculated as a moving Pearson correlation over five-minute windows between 10-second averaged ICP and mean arterial pressure (MAP), updated on a per-minute basis [32, 34, 35]. CPP was defined as MAP minus ICP [32]. CPPopt was estimated minute-by-minute using the method described by Beqiri et al. [5], which utilizes a multi-window weighted analysis of PRx-CPP relationships over time spans ranging from two to eight-hours. In summary, for each data window, preprocessed CPP and Fisher transformed PRx values were grouped into bins, and a second-degree polynomial curve was fitted to the data to identify the nadir (CPPopt). Lastly, quality assurance and averaging were applied, and the resulting estimates were combined with a weighted average and then smoothed using a two-hour exponentially weighted filter [5]. Minute-by-minute CPPopt could be calculated for approximately 85% of available CPP data, consistent with previously reported yield values [5, 36]. ΔCPPopt was defined as the difference between actual CPP and CPPopt (ΔCPPopt = CPP_actual_ – CPPopt) [7]. All physiological variables were evaluated on a minute-by-minute scale for subsequent analysis.

### Cerebral physiological analyses

Cerebral physiological analyses were conducted during the first seven days post-injury. Data acquisition was occasionally interrupted when patients left the intensive care unit (ICU), e.g., due to imaging or surgery. After excluding missing data including artifactual periods, the remaining monitoring time was defined as the percentage of good monitoring time (%GMT). Median values of ICP, PRx, CPP, and CPPopt were calculated for this period. Additionally, each variable’s %GMT within specific thresholds was calculated as a descriptor of insult burden: ICP above 22 mmHg (classified as an insult per Brain Trauma Foundation [BTF] guidelines [31]), PRx above 0.2 (threshold suggested to indicate the beginning of CPA impairment [5, 37, 38]), CPP below/within/above 60–70 mmHg (hypoperfusion/normal/potential hyperemia ranges respectively according to the BTF guidelines [31, 37]), and ΔCPPopt below/within/above ± 5 mmHg (hypoperfusion/adequate perfusion/hyperperfusion ranges respectively as extrapolated from the COGiTATE trial protocol [6]).

### Clinical outcome

Clinical outcome was evaluated with GOSE at six months post-injury, which categorizes outcomes from death (1) to upper good recovery (8) [39, 40], and was dichotomized into favorable/unfavorable (GOSE 5–8/1–4) and mortality/survival (GOSE 1/2–8).

### Statistical analysis

All data analyses were limited to the first seven days post-injury. Demographic data were presented as medians (interquartile range [IQR]) for continuous variables and as numbers (percentage [%]) for categorical variables. Custom written R-scripts were used for visualization of the data and detailed descriptions are available in the supplemental methods.

To illustrate the temporal data frequency, scatterplots were generated displaying log10-transformed PBBM levels and daily medians of cerebral physiological variables, with temporal trends visualized as local regression with locally estimated scatterplot smoothing (LOESS).

To explore the general association between PBBMs and cerebral physiological variables, a grid cell heatmap of Spearman correlations between median values for the first seven days of each pair was generated. Furthermore, the same principle was used to explore the association of %GMT outside/within certain cerebral physiological thresholds with PBBM concentrations. We also examined whether day 1 PBBM levels reflected the cumulative burden of cerebral physiological disturbances over day 1–7. The Spearman correlation coefficients were color-coded from negative to positive, scaled from − 0.5 to 0.5. Furthermore, we evaluated the presence of a secondary PBBM peak using an exploratory definition as a ≥ 10% increase between consecutive samples preceded by a decrease by any magnitude in patients with at least four consecutive PBBM measurements, to see if it was associated with differences in the %GMT of cerebral physiological insults between patients with and without a secondary peak using Mann Whitney U-test.

To capture the temporal dynamics between PBBMs and cerebral physiological variables, a cross correlation plot was constructed with lags of 24-hours. To obtain one value, the median was calculated per patient, per day, and per lag. All PBBM values (per patient, per day) were matched with the corresponding physiological values (per patient, per day, and per lag). For correlation calculations, Spearman’s correlation analysis was used, and 95% confidence interval (CI) of the correlation coefficients were determined for each time lag through bootstrapping with 1000 iterations. The time lags of 24-hours represent a shift of cerebral physiological variables in relation to PBBM collection. The Spearman’s correlation coefficient was then plotted with 95% CI across the time lags. The same principle was applied to visualize predefined time windows of median cerebral physiological data in closer temporal proximity (0.5, 1, 2, and 6 h) before and after collection of PBBM in correlation to PBBM concentration.

To examine if PBBMs varied in correlation to cerebral physiology, a univariate linear mixed effects model (LMEM) was fitted for each PBBM, further, to adjust for repeated measures, patient was used as a random intercept. Data gathered during the first seven days post-injury was used and the median for each cerebral physiological variables per day was calculated. From this, scatterplots were created with log10-transformed PBBM levels and cerebral physiological variables, overlaid with model fits and data density. A multivariate LMEM was used to assess the independent effects of cerebral physiological variables on each PBBM with ICP, PRx, age, GCS motor (GCS M) score at admission and either CPP or ΔCPPopt as fixed effects. Interactions between PRx and CPP/ΔCPPopt or ICP were included based on previous studies [41]. Since ABP variability theoretically can attenuate ΔCPPopt-insults, an additional multivariate LMEM including ΔCPPopt_negative_ and ΔCPPopt_positive_ was done – reflecting hypo- and hyperperfusion. For ΔCPPopt_negative_ all minute values of ΔCPPopt > 0 mmHg were counted as zeroes and the absolute values were used for values < 0 mmHg (e.g., CPP at 65 mmHg and CPPopt at 75 mmHg yielded a ΔCPPopt_negative_ of 10 mmHg). Similarly, for ΔCPPopt_positive_ all values < 0 mmHg of ΔCPPopt were counted as zeroes. In both multivariate LMEMs, patient and time (days) were modeled as random effects with patient as a grouping factor for random effects, with both a random intercept and a random slope for time (days), allowing each patient to have an individual baseline and a patient-specific trajectory over the observation period.

Benjamini-Hochberg (BH) correction was applied to control false discovery rate across multiple simultaneous comparisons with adjusted *p* values reported as *q* values. A *p* and *q* value < 0.05 were considered statistically significant. All statistical analyses were conducted using the software R, version 4.3.2 (R Core Team, R Foundation for Statistical Computing, Vienna, Austria).

## Results

### Demographic data

The study included 151 patients (Table 1), with a median age of 52 years (IQR 31–64). The most common causes of injury were road traffic incident (45%) and incidental fall (34%). The median GCS M was 4 (IQR 1–5) and 34 (23%) patients exhibited one or two unreactive pupils at admission. The median Marshall grade was 3 (IQR 2–6) and median ISS was 34 (IQR 25–45). Traumatic subarachnoid hemorrhage (76%), contusions (63%), and acute subdural hematomas (51%) were more common radiological findings, whereas intraventricular hemorrhage (29%) and epidural hematoma (21%) were less frequent. At six-month follow-up, 71 patients (47%) had recovered favorably, while 32 patients (21%) were deceased.

**Table 1:** Demographic data

Variable	
**Patients**, ***n (%)***	151 (100%)
**Age**,** median (IQR) years**	52 (31–64)
**Cause of injury**, ***n*** **(*%*)**	151 (100%)
Road traffic incidents, *n (%)*	68 (45%)
Incidental fall, *n (%)*	51 (34%)
Other non-intentional injury, *n (%)*	6 (4%)
Violence, n (%)	10 (7%)
Suicide attempt, *n (%)*	1 (1%)
Other, *n (%)*	8 (5%)
Unknown, *n (%)*	7 (5%)
**Pupillary status**, ***n (%)***	142 (94%)
Normal, *n (%)*	105 (74%)
Unilateral non-reactive, *n (%)*	9 (6%)
Bilateral non-reactive, *n (%)*	21 (15%)
Unknown, *n (%)*	7 (5%)
**GCS M at admission**,** median (IQR) score**	4 (1–5)
**Total ISS**,** median (IQR) score**	34 (25–45)
**Marshall grade**,** median (IQR) score**	3 (2–6)
**EDH**, ***n (%)***	32 (24%)
**ASDH**, ***n (%)***	77 (57%)
**tSAH**, ***n (%)***	114 (88%)
**IVH**, ***n (%)***	44 (33%)
**Contusion**, ***n (%)***	95 (70%)
**Favorable outcome**, ***n (%)***	71 (47%)
**Mortality**, ***n (%)***	32 (21%)

Missing data: ASDH *n* = 16 (11%), contusion *n* = 16 (11%), EDH *n* = 16 (11%), GCS M at admission *n* = 3 (2%), IVH *n* = 16 (11%), Marshall grade *n* = 23 (15%), pupillary status *n* = 9 (6%), tSAH *n* = 22 (15%)

ASDH = Acute Subdural Hematoma. EDH = Epidural Hematoma. Favorable outcome = GOSE 5–8 at 6-months. GCS M = Glasgow Coma Scale Motor score. ISS = Injury Severity Score. IVH = Intraventricular Hemorrhage. Mortality = GOSE 1 at 6-months. tSAH = traumatic subarachnoid hemorrhage. IQR = Interquartile Range

### Temporal dynamics of proteomic blood-based biomarkers and cerebral physiological variables

The median values (IQR) for PBBMs and cerebral physiological variables during the first seven days post-injury, as well as %GMT outside/within the predefined intervals for cerebral physiological variables are summarized in Table 2. The data density of PBBMs and cerebral physiology variables are illustrated in Supplemental Fig. 2. For all data, the highest density was noted for day 1. For GFAP, NSE, S100B, t-Tau, and UCH-L1 a decrease in median values was noted, whilst a gradual increase was noted for NfL (Supplemental Fig. 2a). The median value for the cerebral physiological variables stayed fairly consistent throughout the examined period (Supplemental Fig. 2b).

**Table 2:** Proteomic blood-based biomarkers and cerebral physiological data during the first seven days following injury

Variable	
Patients, n *(%)*	151 *(100%)*
*Proteomic blood-based biomarkers*	
GFAP (ng/mL), median (IQR)	15 (4–38)
NfL (pg/mL), median (IQR)	98 (54–192)
NSE (ng/mL), median (IQR)	15 (10–24)
S100B (µg/L), median (IQR)	0.13 (0.07–0.31)
t-Tau (pg/mL), median (IQR)	5 (2–13)
UCH-L1 (pg/mL), median (IQR)	206 (83–551)
*Cerebral physiology*	
ICP (mmHg), median (IQR)	12 (9–15)
ICP > 22 mmHg (%GMT), median (IQR)	3 (1–7)
PRx, median (IQR)	0.00 (-0.13-0.14)
PRx > 0.2 (%GMT), median (IQR)	30 (21–44)
CPP (mmHg), median (IQR)	70 (63–77)
CPP < 60 mmHg (%GMT), median (IQR)	14 (3–36)
CPP 60–70 mmHg (%GMT), median (IQR)	29 (16–37)
CPP > 70 mmHg (%GMT), median (IQR)	50 (24–74)
CPPopt (mmHg), median (IQR)	70 (65–77)
ΔCPPopt (mmHg), median (IQR)	0 (-3-1)
ΔCPPopt < -5 mmHg (%GMT), median (IQR)	31 (22–41)
ΔCPPopt ± 5 mmHg (%GMT), median (IQR)	38 (33–46)
ΔCPPopt > 5 mmHg (%GMT), median (IQR)	27 (18–36)

GFAP = Glial Fibrillary Acidic Protein. NfL = Neurofilament Light Chain. NSE = Neuron-Specific Enolase. S100B = S100 calcium-binding protein B. t-Tau = total Tubulin associated unit. UCH-L1 = Ubiquitin C-terminal Hydrolase-L1. CPP = Cerebral Perfusion Pressure. CPPopt = Optimal CPP. ΔCPPopt = CPP – optimal CPP. GMT = Good Monitoring Time. ICP = Intracranial Cerebral Pressure. PRx = Pressure Reactivity Index. IQR = Interquartile Range

### The association between proteomic blood-based biomarkers and cerebral physiological variables – an exploratory Spearman correlation analysis of seven days-median values

**Fig. 1:**
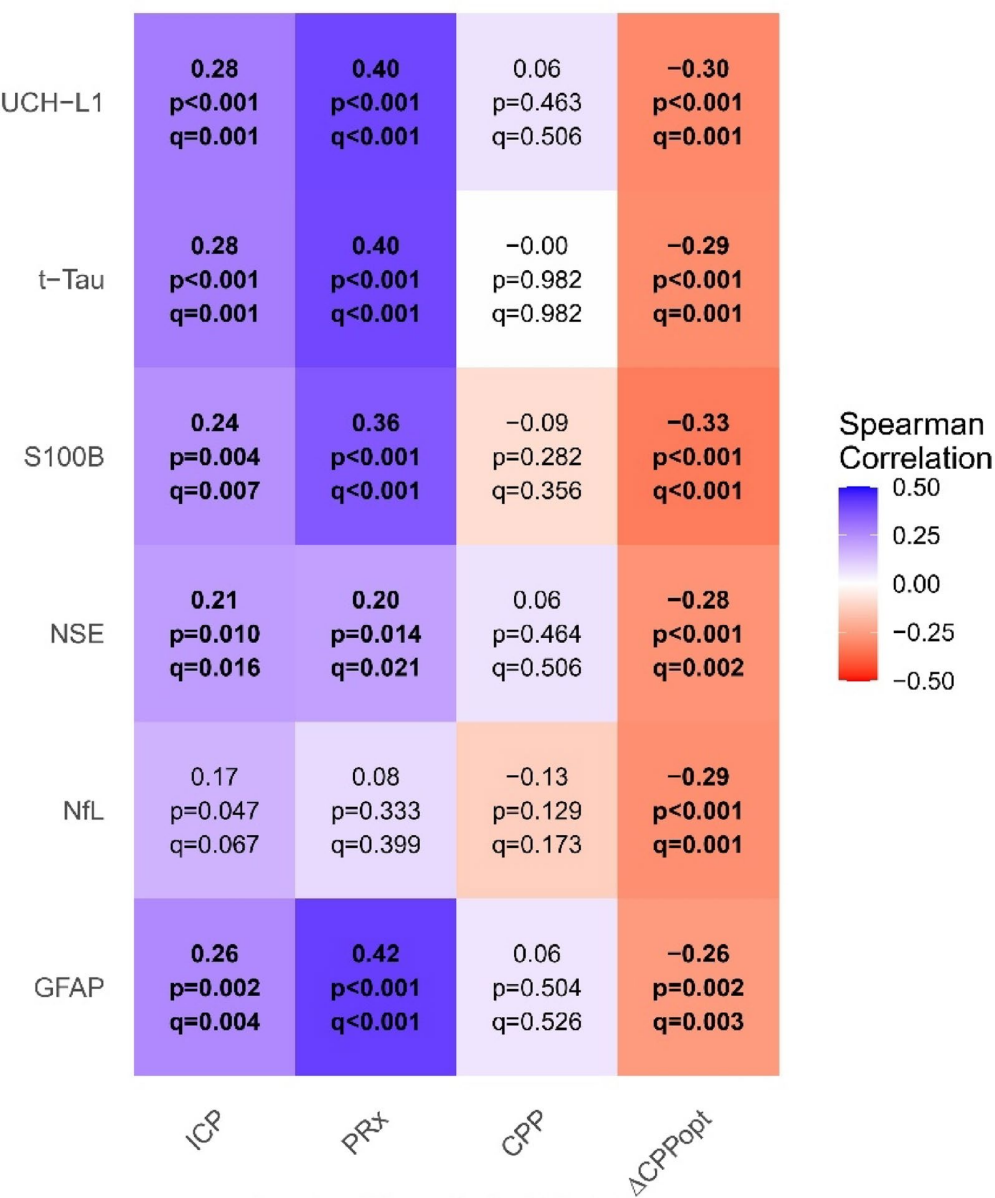
The association between proteomic blood-based biomarkers and cerebral physiological variables – A Spearman correlation matrix of data gathered during the first seven days post-injury. Correlation matrix between PBBMs (GFAP, NfL, NSE, S100B, t-Tau, and UCH-L1) and cerebral physiological variables (ICP, PRx, CPP, and ΔCPPopt) during the first week using Spearman correlation analysis. To illustrate the correlation coefficient, grid cells are color-coded from red (negative) to blue (positive). Statistically significant values (*p* and *q* < 0.05) are bolded. A positive and statistically significant correlation was seen for GFAP and ICP, PRx; for NSE and ICP, PRx; for S100B and ICP, PRx; for t-Tau and ICP, PRx; for UCH-L1 and ICP, PRx; meanwhile a negative correlation was seen for all PBBMs and ΔCPPopt

The first exploratory analysis investigated the correlation between median levels of PBBMs and cerebral physiological variables gathered during the first seven days post-injury, and is illustrated in Fig. 1. Higher ICP levels demonstrated a statistically significant correlation with elevated levels of all PBBMs. Higher PRx correlated significantly with higher levels of GFAP (*p* < 0.001), NSE (*p* = 0.014), S100B (*p* = 0.001), t-Tau (*p* < 0.001), and UCH-L1 (*p* < 0.001). CPP did not show any significant correlation with PBBMs. Higher ΔCPPopt demonstrated significant correlation with lower levels of all PBBMs. Notably, only the NfL correlation with ICP did not remain statistically significant following BH correction. Additionally, %GMT of cerebral physiological variables within predefined ranges correlated with PBBM concentration during the first seven days post-injury (Supplemental Fig. 3). Higher levels of all PBBMs on day 1 correlated with higher %GMT of ICP > 22 mmHg insults during the first seven days, while higher day 1-levels of GFAP, t-Tau, and UCH-L1 were associated with higher %GMT of PRx > 0.2 insults and inversely with lower %GMT CPP 60–70 mmHg (Supplemental Fig. 4). Elevated day 1-levels of all PBBMs correlated with higher %GMT ΔCPPopt < -5 mmHg, while higher day 1-levels of all PBBMs were associated with lower %GMT ΔCPPopt > 5 mmHg. All of these correlations remained statistically significant after BH correction except the correlation between UCH-L1 and %GMT PRx > 0.2.

Whether the burden of %GMT insults differed during the first seven days post-injury between patients with and without secondary PBBM peaks (defined as ≥ 10% increase between consecutive samples preceded by a decrease by any magnitude and ≥ 4 consecutive sampling days; Supplemental Table 1) was also analyzed. A secondary peak of GFAP seemed to correlate significantly with higher median ICP (*p* = 0.016). Patients with a secondary t-Tau peak exhibited a higher proportion of %GMT CPP < 60 mmHg (*p* = 0.028) and a lower proportion of %GMT CPP > 70 mmHg (*p* = 0.033) and correlated to lower median CPP (*p* = 0.044). A secondary peak of UCH-L1 correlated significantly with lower median CPPopt (*p* = 0.026). Importantly, after BH correction no correlation stayed statistically significant.

### Cross correlation analysis of proteomic blood-based biomarkers vs. cerebral physiological variables across the first seven days post-injury

**Fig. 2:**
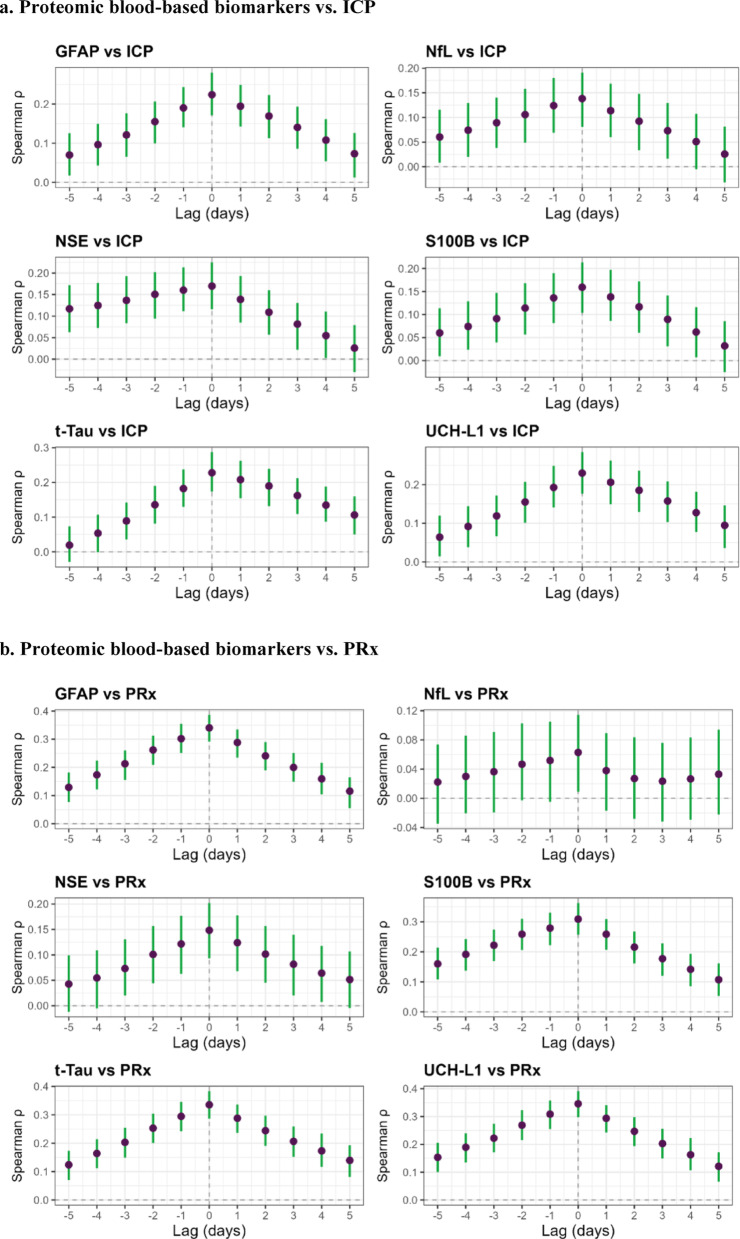

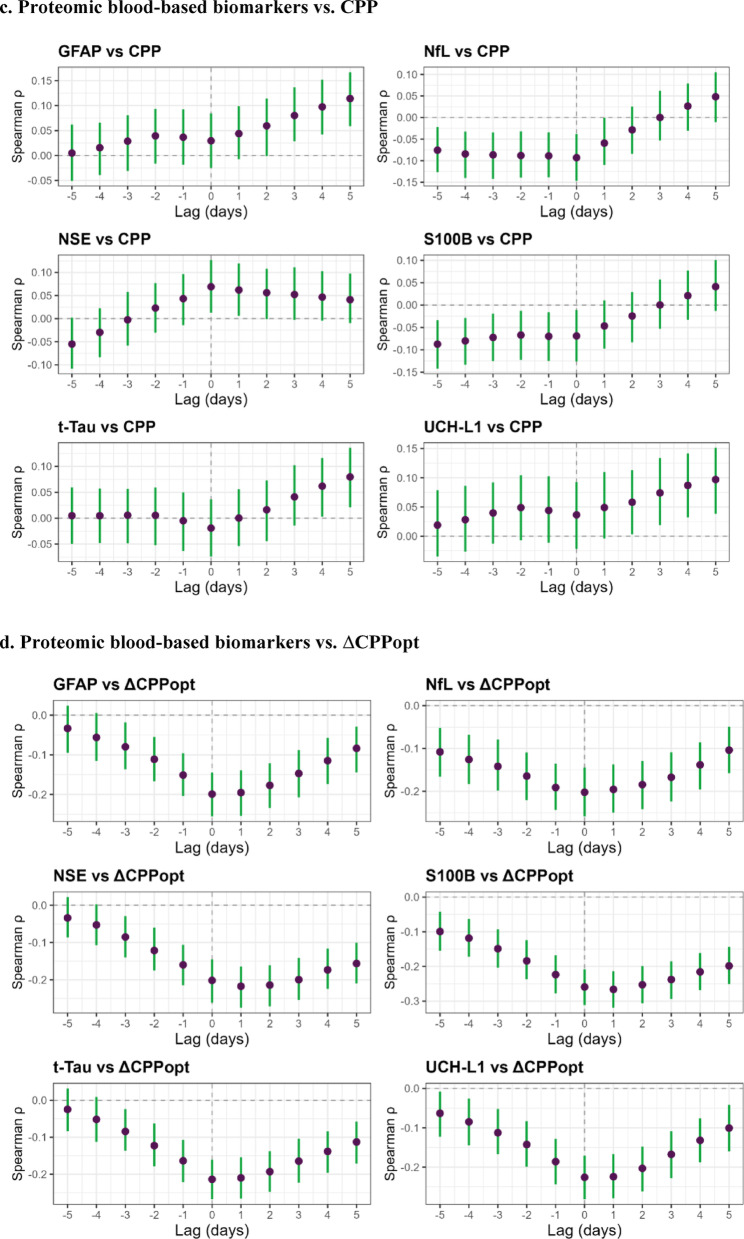
Proteomic blood-based biomarkers vs. Cerebral physiological variables – 24-hour lag cross correlation. Cross correlation plot demonstrating the Spearman correlation coefficient with 95% CI between PBBM concentration (GFAP, NfL, NSE, S100B, t-Tau, and UCH-L1) and cerebral physiological variables (ICP, PRx, CPP, and ΔCPPopt). The lags were set to a 24-hour period shift of cerebral physiological variables in relation to PBBM collection. As visualized, both ICP and PRx demonstrated the highest positive correlation with lag 0 for all PBBMs. CPP had generally negative correlation for negative lags and positive correlation for positive lags for all PBBMs. ΔCPPopt displayed the most negative correlation with lag 0 or 1 for all PBBMs

The temporal dynamics of the correlations between PBBMs and cerebral physiological variables were examined in cross correlation plots with lags of 24-hours (Fig. 2). These correlations with PBBM levels were used to explore the temporal association in the relation between physiological insults and PBBM concentration. In summary, for all PBBMs, the strongest correlation with all cerebral physiological variables was with lag 0 – for values gathered the same day. Additionally, shorter time windows of 0.5, 1, 2, and 6 h before and after PBBM sampling was used for a more temporally narrow examination of correlations (Supplemental Fig. 5). Overall, the correlation coefficients were relatively consistent across all shorter time windows.

### Proteomic blood-based biomarker vs. Cerebral physiological variables – A univariate linear mixed effects model analysis

**Fig. 3:**
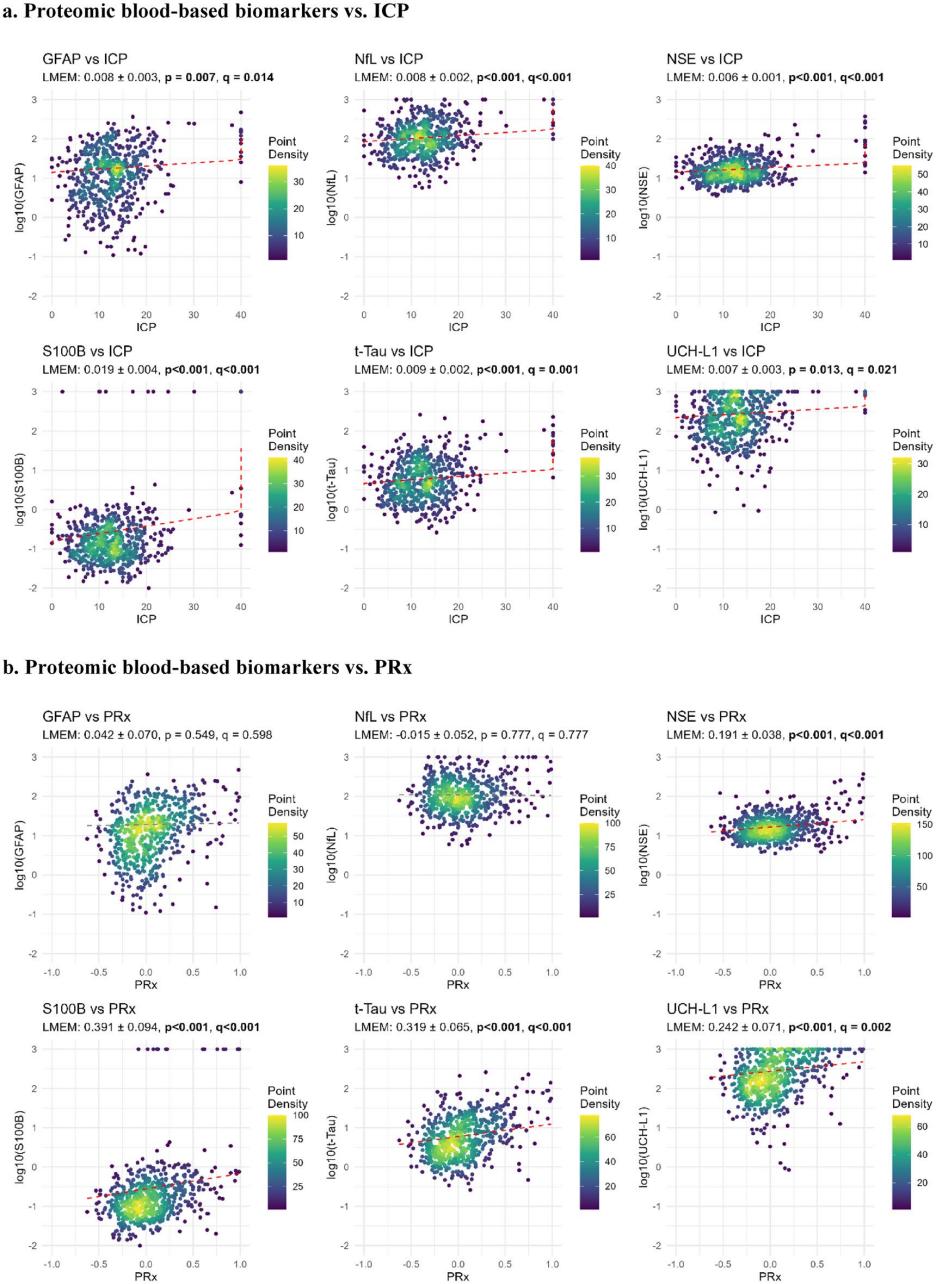

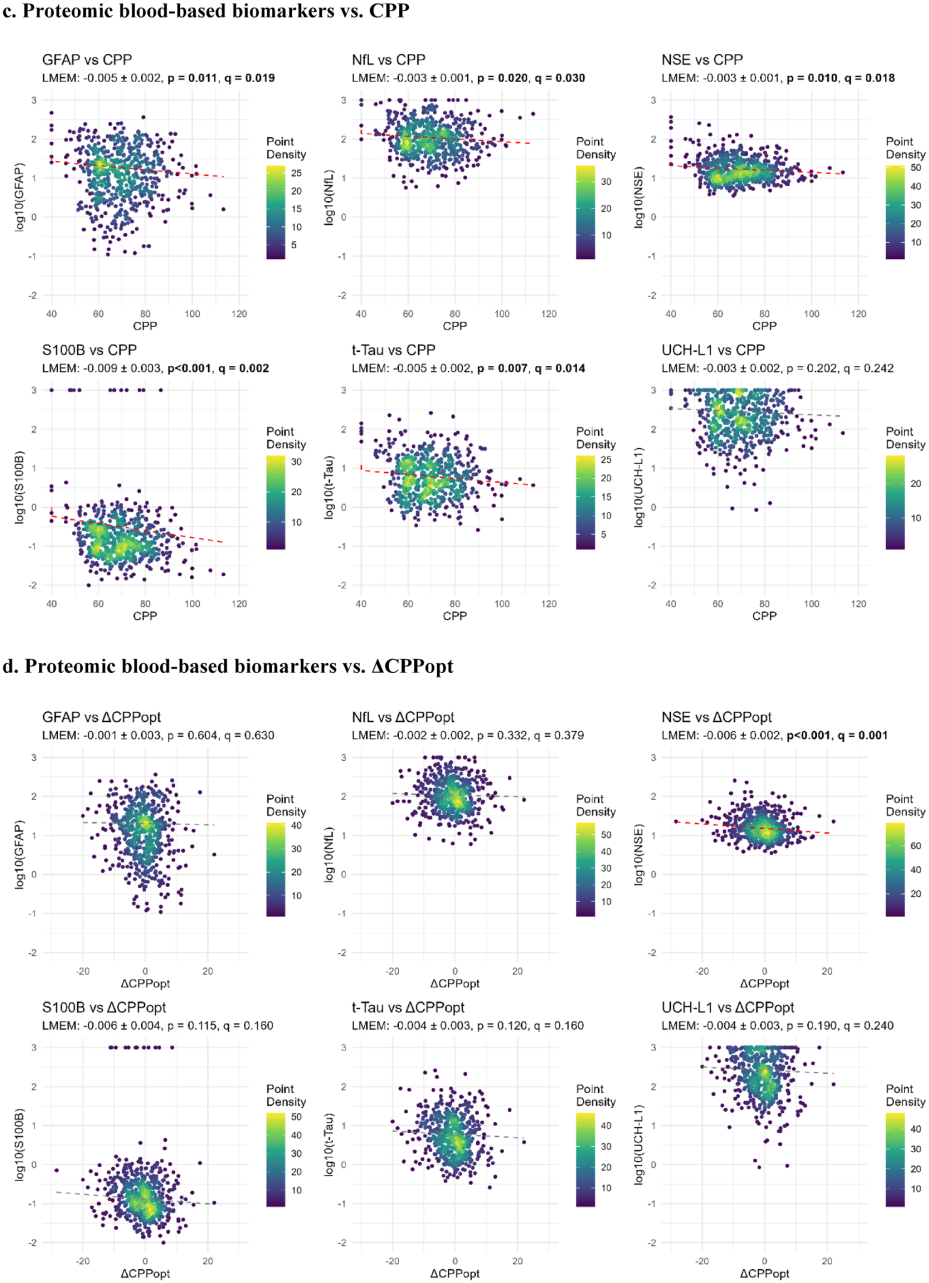
Proteomic blood-based biomarkers vs. Cerebral physiological variables – A univariate linear mixed effects model. Scatterplots including LMEM visualizing GFAP, NfL, NSE, S100B, t-Tau, and UCH-L1 in relation to ICP, PRx, CPP, and ΔCPPopt during first seven days post-injury using univariate LMEM models (dashed red line). *Oob squish* function was used to visualize outliers on the outermost perimeter. Data density was visualized through the viridis color-scale ranging from blue (low) to yellow (high). As visualized, a statistically significant univariate model was seen for the combinations GFAP with ICP and CPP; NfL with ICP and CPP; NSE with ICP, PRx, CPP and ΔCPPopt; S100B with ICP, PRx, and CPP; t-Tau with ICP, PRx, and CPP; UCH-L1 with ICP and PRx. All results remained statistically significant following Benjamini-Hochberg correction (*q* < 0.05)

Figure 3 displays a LMEM for all PBBMs against 24 h medians of cerebral physiological variables. Higher ICP displayed statistically significant correlation with elevated levels of all PBBMs. For PRx, a positive slope was seen for all PBBMs except GFAP and NfL. Statistical significance was noted for higher CPP in correlation with lower levels of GFAP (*p* < 0.05), NfL (*p* < 0.05), NSE (*p* = 0.01), S100B (*p* < 0.001), and t-Tau (*p* < 0.01). Lastly, positive ΔCPPopt showed a statistically significant correlation with lower NSE (*p* < 0.001), but not with any other PBBM. All correlations remained statistically significant after BH was applied.

In multivariate LMEMs (Table 3), each PBBM was analyzed in separate LMEMs containing ICP, PRx, and either CPP or ΔCPPopt (in separate models) together with age and GCS M at admission as fixed effects, further with patient and time (days) as random effects. The interaction between PRx vs. ICP and CPP/ΔCPPopt were also evaluated in these models. Higher ICP was independently associated with higher levels of each PBBM in both of the multivariate LMEMs. Higher PRx was associated with lower NfL in both models. Higher ΔCPPopt correlated with lower NSE. A significant positive interaction between ICP and PRx was observed in both models for NSE, as well as for NfL in the ΔCPPopt model and S100B in the CPP model. Moreover, the interaction between PRx and ΔCPPopt was positive and significant in the model for S100B. Lastly, age correlated positively and significantly with GFAP, S100B, t-Tau, and UCH-L1, as well as negatively with NSE in both models. Additionally, GCS M at admission was significantly and negatively correlated with both NfL and NSE models. Following BH correction PRx lost its statistical significance in the NfL model. This was also noted for ICP, age, ΔCPPopt, and the interaction between ICP and PRx in the NSE models. The multivariate LMEM with ΔCPPopt_negative_ and ΔCPPopt_positive_ was also examined (Supplemental Table 2). Higher ICP was significantly associated with higher levels of each PBBM in both models. PRx was significant in the NfL (negatively) and S100B (positively) models. ΔCPPopt_negative_ was statistically significant in the NSE model (positively). The interaction between ICP and PRx demonstrated a significant positive correlation in the NfL and NSE model, while the interaction between PRx and ΔCPPopt_negative_ was significant in the S100B (negatively) model. Age was significant in all models except the NfL model (positively in all models except the NSE models), and GCS M at admission was significant in the NfL and NSE models (negatively in both). Following BH correction, the NSE model lost statistical significance with ΔCPPopt_negative_, age, and the interaction between ICP and PRx. The S100B model lost statistical significance with PRx, and the t-Tau model with age.

**Table 3:** Proteomic blood-based biomarkers vs. Cerebral physiological variables during the first seven days post-injury – A multivariate linear mixed effects model analysis

Variable	GFAP			NfL			NSE			S100B			t-Tau			UCH-L1		
	Value ± SE	*p* value	q value	Value ± SE	*p* value	q value	Value ± SE	*p* value	q value	Value ± SE	*p* value	q value	Value ± SE	*p* value	q value	Value ± SE	*p* value	q value
ICP	0.022 ± 0.004	**< 0.001**	**< 0.001**	0.012 ± 0.003	**< 0.001**	**0.003**	0.004 ± 0.002	**0.037**	0.104	0.026 ± 0.005	**< 0.001**	**< 0.001**	0.013 ± 0.004	**0.001**	**0.007**	0.015 ± 0.004	**< 0.001**	**0.005**
PRx	-0.215 ± 0.417	0.606	0.710	-0.636 ± 0.313	**0.043**	0.112	0.062 ± 0.245	0.800	0.861	-0.290 ± 0.595	0.626	0.710	0.706 ± 0.407	0.083	0.189	0.287 ± 0.453	0.527	0.663
CPP	-0.003 ± 0.002	0.110	0.215	-0.002 ± 0.002	0.266	0.407	-0.001 ± 0.001	0.572	0.696	-0.002 ± 0.003	0.405	0.549	-0.001 ± 0.002	0.507	0.663	0.000 ± 0.002	0.957	0.957
ICP: PRx	-0.013 ± 0.008	0.095	0.196	0.008 ± 0.005	0.127	0.234	0.008 ± 0.004	**0.035**	0.102	0.019 ± 0.010	**0.050**	0.125	-0.004 ± 0.007	0.568	0.696	-0.001 ± 0.008	0.217	0.350
PRx: CPP	0.005 ± 0.005	0.389	0.548	0.007 ± 0.004	0.095	0.196	0.000 ± 0.003	0.939	0.957	0.005 ± 0.007	0.516	0.663	-0.006 ± 0.005	0.244	0.386	0.000 ± 0.006	0.946	0.957
Age	0.010 ± 0.002	**< 0.001**	**0.001**	-0.000 ± 0.002	0.831	0.884	-0.002 ± 0.001	**0.042**	0.112	0.007 ± 0.002	**0.003**	**0.013**	0.004 ± 0.002	**0.009**	**0.034**	0.005 ± 0.002	**0.010**	**0.035**
GCS M at admission	-0.037 ± 0.024	0.124	0.234	-0.049 ± 0.016	**0.003**	**0.013**	-0.029 ± 0.008	**< 0.001**	**0.004**	-0.020 ± 0.023	0.402	0.549	-0.028 ± 0.016	0.096	0.196	-0.035 ± 0.019	0.073	0.171
ICP	0.022 ± 0.004	**< 0.001**	**< 0.001**	0.010 ± 0.003	**0.001**	**0.007**	0.006 ± 0.002	**0.003**	**0.013**	0.026 ± 0.005	**< 0.001**	**< 0.001**	0.012 ± 0.004	**0.002**	**0.011**	0.014 ± 0.005	**0.002**	**0.012**
PRx	0.102 ± 0.133	0.444	0.593	-0.317 ± 0.090	**< 0.001**	**0.004**	0.058 ± 0.067	0.391	0.548	0.289 ± 0.168	0.086	0.190	0.081 ± 0.128	0.529	0.663	0.153 ± 0.141	0.278	0.410
ΔCPPopt	0.001 ± 0.003	0.861	0.904	0.001 ± 0.002	0.642	0.719	-0.004 ± 0.002	**0.020**	0.059	-0.008 ± 0.004	0.073	0.171	-0.002 ± 0.003	0.599	0.710	-0.002 ± 0.003	0.622	0.710
ICP: PRx	-0.011 ± 0.009	0.214	0.350	0.020 ± 0.006	**0.001**	**0.007**	0.010 ± 0.004	**0.018**	0.057	0.005 ± 0.011	0.614	0.710	0.010 ± 0.009	0.275	0.410	-0.001 ± 0.009	0.900	0.934
PRx:ΔCPPopt	-0.014 ± 0.009	0.100	0.201	-0.009 ± 0.006	0.145	0.259	0.002 ± 0.005	0.737	0.804	0.036 ± 0.012	**0.004**	**0.013**	-0.012 ± 0.009	0.173	0.297	-0.011 ± 0.010	0.267	0.407
Age	0.009 ± 0.002	**< 0.001**	**0.002**	-0.001 ± 0.002	0.679	0.750	-0.002 ± 0.001	**0.047**	0.118	0.008 ± 0.002	**0.002**	**0.011**	0.004 ± 0.002	**0.015**	**0.048**	0.005 ± 0.002	**0.010**	**0.035**
GCS M at admission	-0.034 ± 0.025	0.172	0.297	-0.049 ± 0.016	**0.003**	**0.013**	-0.030 ± 0.008	**< 0.001**	**0.004**	-0.025 ± 0.024	0.309	0.447	-0.023 ± 0.017	0.178	0.299	-0.031 ± 0.020	0.128	0.234

Based on the cerebral physiological variables, two separate models including interactions were used to assess the independent effect on each PBBM. The models included ICP, PRx, CPP or ΔCPPopt, the interaction terms ICP: PRx and PRx: CPP or PRx:ΔCPPopt, as well as age and GCS M at admission. Random effects were patients and time (days). All *p* and *q* values of statistical significance (< 0.05) are bolded. GFAP = Glial Fibrillary Acidic Protein. NfL = Neurofilament Light Chain. NSE = Neuron-Specific Enolase. S100B = S100 calcium-binding protein B. t-Tau = total Tubulin associated unit. UCH-L1 = Ubiquitin C-terminal Hydrolase-L1. CPP = Cerebral Perfusion Pressure. CPPopt = Optimal CPP. ΔCPPopt = CPP – optimal CPP. ICP = Intracranial Cerebral Pressure. PRx = Pressure Reactivity Index. SE = Standard Error

## Discussion

In this observational CENTER-TBI study of 151 TBI patients with serial measurements of an extensive PBBM panel and high-frequency cerebral physiological monitoring, we confirmed our hypothesis that PBBM levels were associated with key cerebral physiological variables. PBBM concentrations on day 1 were associated with the subsequent trends of ICP, PRx, and ΔCPPopt defined insults over the following seven days post-injury. Median PBBM levels throughout the monitoring period correlated with a higher cumulative burden of these cerebral physiological disturbances. Temporal analyses indicated that the strength of these associations was the strongest when examining cerebral physiological variables and PBBM concentrations gathered the same day, and the associations are likely bidirectional: early PBBM surges reflect a primary injury burden that predisposes to adverse physiological trajectories, while secondary cerebral physiological insults may in turn drive further PBBM release – though no causal directionality can be inferred from these observational data. In multivariate LMEMs, several cerebral physiological variables were independently associated with multiple PBBMs, with elevated ICP showing the most consistent and robust associations. Other cerebral physiological parameters demonstrated weaker relationships, but impaired CPA (high PRx) tended to potentiate the PBBM effects of increased ICP. Finally, no single PBBM emerged as clearly superior in reflecting cerebral physiological or CPA disturbances; rather, NfL appeared less responsive, likely due to its different release and clearance kinetics [13, 23–25].

Day 1 PBBM levels have previously been shown to reflect the extent of primary brain injury, correlate with clinical severity, and correspond to structural damage on radiological imaging in TBI [12, 42]. They have also demonstrated additive value for outcome prediction across different severity strata (GCS groups) [22, 43]. In the present study, we confirm that higher day 1 PBBM levels were associated with a higher burden of ICP and PRx insults, and ΔCPPopt disturbances. This suggests that early PBBM levels may help identify patients at risk of an unfavorable intensive care trajectory and the potential need for timely interventions to prevent additional secondary insults and injury progression. We further observed that median PBBM levels over the first seven days correlated with the corresponding 7-day medians of cerebral physiological variables. Although most PBBMs, except NfL, declined after day 1, and delayed clearance or secondary peaks may reflect ongoing or secondary cerebral insults [44, 45]. For this reason, we explored these temporal relationships in greater detail.

In univariate LMEMs, higher ICP and PRx, as well as lower CPP and negative ΔCPPopt values over time, were frequently associated with higher levels of all PBBMs – except for NfL with PRx. To aid clinical interpretation of the LMEM effect sizes (Table 3), all PBBM values are log10-transformed, thus, the GFAP coefficient for ICP (0.022 per mmHg) implies that a 10 mmHg increase in daily median ICP is associated with approximately a 1.66-fold increase in GFAP concentration (10^[0.022 × 10]^ ≈ 1.66). Similar translations apply to other PBBM–cerebral physiological variable pairs (Table 3). When examining different temporal offsets between the cerebral physiological variables and PBBM sampling, the strength of the correlation coefficients was generally higher for cerebral physiological variables and PBBM gathered during the same 24-hour period. As previously stated, no assumptions were made regarding the directionality or causality of these relationships. Patients with more extensive underlying primary brain injury typically release more PBBM and, as a consequence of the injury, develop more cerebral edema, and episodes of impaired CPA [10–12, 15–17, 19]. In turn, these secondary physiological insults may further exacerbate tissue damage and PBBM release, creating a detrimental positive feedback loop. The overall similarity in correlation strengths across different time windows may reflect the slow kinetics of PBBM release and clearance. Accordingly, these data are consistent with bidirectional associations – but no directional or causal conclusions can be drawn from this exploratory study. We explicitly refrain from interpreting the temporal lags as evidence of causation [44]. Moreover, although cerebral physiological insults can be transient, their underlying contributors, such as brain edema, extent of intracranial bleedings, and the patient’s baseline blood pressure, typically remain relatively stable at least when evaluated over a few hours. Thus, patients who exhibited such acute disturbances prior to PBBM sampling were likely to continue experiencing them thereafter, reinforcing a certain temporal stability of the observed correlations. The additional analysis of secondary peaks with a definition of 10% increase between two subsequent PBBM values preceded by a decrease of any magnitude and ≥ 4 consecutive sampling days, displayed a few statistically significant results with more cerebral physiological insults, however, none of the observed significant results were persistent after BH correction. Furthermore, the term secondary peak is not clearly defined for each PBBM, where different frequencies of PBBM sampling and clearance time complicated the analysis. The threshold of 10% is arbitrary and does not account for PBBM-specific clearance kinetics; the requirement for ≥ 4 consecutive sampling days may also introduce selection bias toward patients with less complicated ICU courses. This analysis should therefore be considered purely exploratory. Additionally, a recent study demonstrated that trajectories with consistently elevated PBBMs rather than secondary peaks were linked to worse outcome [45]. Future research should aim at investigating PBBM data gathered several times per day and explore different interpretations of secondary peaks in regards to individual PBBM clearance and trajectories.

In the multivariate analyses, ICP was the strongest cerebral physiological correlate, showing associations with most PBBMs, except NSE following BH correction. Our results confirm and extend prior work [10, 11] demonstrating that ICP elevations are associated with both astrocytic (GFAP, S100B) and neuronal/axonal injury (UCH-L1, NfL, t-Tau) following TBI. ICP, in particular, reflects the underlying injury burden itself, which may explain its dominant role [46–48]. In addition, critically elevated ICP is known to have catastrophic effects on cerebral integrity and outcome [46–48], further supporting its strong association with the PBBMs. Regarding CPA insults, PRx primarily acted as a modifier, amplifying the deleterious effects of elevated ICP and to some extent ΔCPPopt insults. This interaction pattern is consistent with the notion that impaired CPA does not constitute an insult on its own, but rather increased risk of one, which is also supported by previous outcome studies showing that high PRx narrows the tolerable range of ICP, CPP, and ΔCPPopt associated with favorable outcomes [41, 49]. However, we acknowledge that these results should be interpreted with caution since this was not consistent with all PBBMs and statistical significance was lost after BH correction in some of the models. Furthermore, negative ΔCPPopt, was also associated with higher PBBM levels, emphasizing the contribution of CPA disturbances and hypoperfusion to ongoing secondary injury [50–52]. In these analyses, CPP showed weaker independent effects in the multivariate models, possibly due to the dominant influence of ICP and, perhaps, also the fact that it does not take variations in CPA function into account, which would be consistent with that CPA-guided targets might be more sensitive to cerebral hypo- and hyperperfusion [5, 6, 37, 38]. Altogether, our findings reinforce that PBBMs hold promise both as indicators of secondary brain injury burden in the ICU and as candidate endpoints for CPA-guided trials. We emphasize that their immediate clinical value lies in enriching multimodal neuromonitoring and supporting individualized, CPA-guided care. The findings support a close cerebral physiological–biochemical coupling, suggesting the potential of these PBBMs as candidate endpoints for treatment optimization in the ICU. Given the well-established long-term prognostic value of PBBMs, their apparent responsiveness to acute cerebral physiological stress further underscores their potential clinical relevance for these purposes [10–12, 15–17, 19]. It should be noted that surrogate endpoint validation in the strict sense requires evidence that the PBBM lies on the casual pathway between intervention and clinical outcome, and that treatment-induced changes in the PBBM reliably predict clinical benefit [55].The present observational study does not address these criteria. Rather, our findings position PBBMs as biologically plausible candidate endpoints whose validity should be prospectively tested in CPA-guided trials.

When examining the relationships between cerebral physiological insults and individual PBBMs, no major differences among PBBMs emerged, apart from NfL, which showed a distinct pattern due to its specific release and clearance kinetics, with levels steadily increasing over time [13, 23–25]. Interestingly, and for unclear reasons, higher PRx was paradoxically associated with lower NfL levels in the multivariate analysis. This should be interpreted with caution and deserves further analysis in future studies. S100B has previously been criticized for potential extracranial contamination in TBI [17, 53, 54], and NSE for its susceptibility to hemolysis-related measurement interference [18], whereas GFAP and UCH-L1 have been proposed as more reliable brain-specific markers [14, 15]. In our cohort, however, we did not observe a clear advantage of any single PBBM. The limited influence of extracranial sources for S100B after the first day, as well as generally good sample handling minimizing hemolysis, may partly explain these findings. Beyond these analytical considerations, the PBBMs reflect different cellular aspects of CNS injury, astroglial (GFAP, S100B), neuronal soma (NSE, UCH-L1), and axonal (NfL, t-Tau) [12–14], yet no specific cellular domain appeared more sensitive to cerebral physiological disturbances. Ideally, future prospective studies on the effects of cerebral physiological variables of these PBBMs should include the entire broad panel, excluding perhaps NfL given its delayed kinetics [13, 23–25]. However, if restricted by economic or logistical factors, S100B may represent a pragmatic choice owing to its simplicity, availability, and relatively low cost [54].

Altogether, functional outcome remains a relatively coarse and multifactorial measure, but is difficult to influence due to diverse factors including patient demography, injury complexity, clinical progression, care trajectories, and reintegration into society. In contrast, PBBMs reflecting astrocytic injury, axonal degeneration, and neuronal cell body damage may provide short-term mechanistic insight and could potentially capture the immediate downstream effects of CPA-targeted interventions more sensitively.

### Methodological considerations

This retrospective, observational study has several strengths, the main one being its multi-center design and the first study with detailed variables and high-frequency physiological data as it provides higher external validity and enhances generalizability compared to single-center studies. It provides a comprehensive view of cellular responses to cerebral physiological disturbances, and vice versa. We proceeded our exploratory results with more granular illustrations, with the use of advanced statistical modeling, such as uni- and multivariate LMEM with interaction terms adjusted for repeated measures and confounding variables, as well as cross correlation plots exploring the possible temporal patterns over the most acute phase post-injury. In addition to advanced statistical modeling, all analyses of multiple simultaneous comparisons were corrected using BH method. Limitations must also be acknowledged. First, this was a retrospective, observational multi-center study, using prospectively collected data which precludes causal inference. Second, despite being multi-center, the cohort was moderately sized. Third, collection of PBBM occurred once daily, which introduces a fundamental temporal resolution mismatch with the minute-by-minute cerebral physiological data and may obscure rapid PBBM dynamics in response to transient physiological insults, limiting the strength of temporal inferences. Future studies should explore more frequent PBBM sampling. Fourth, most patients had multiple PBBM samples, but such data were scarcer during the last days of the first week. Fifth, to acknowledge the PBBMs different clearance times, ranging from hours to days, we chose to explore certain time windows but recognize that future studies should investigate additional time windows. Furthermore, CPPopt is influenced by past data up to 8 h of ICP and ABP measurement, making it less responsive to the shorter time windows. Sixth, we also acknowledge that there is an interplay among overall and baseline PBBM levels, as well as secondary peaks and persistent PBBM elevations that may affect possible correlation to cerebral physiology. The lack of analytical frame-works capable of separating these components limits causal interpretation and should be considered when evaluating associations between PBBMs and cerebral physiological variables, hence, it should be further investigated. Seventh, clinical PBBM analysis by the platform Roche was used for NSE and S100B, which has a lower coefficient of variation than the platform Quanterix used for GFAP, NfL, t-Tau, and UCH-L1. This could potentially have blunted the correlations of the analyzed PBBMs. Lastly, analyses were not stratified by TBI subtype. Given the heterogeneity of TBI, the observed associations between PBBMs and cerebral physiological variables may vary across different injury phenotypes (focal vs. diffuse, severity etc.). The CENTER-TBI cohort encompasses a broad spectrum of injury severity, mechanism, and radiological pattern (including focal vs. diffuse injury), which may have introduced heterogeneity that dilutes subgroup-specific associations. The present study was not powered to explore such differences, although, GCS M was included as a fixed effect in the multivariate LMEM analyses, providing partial adjustment for severity. Hence, future adequately powered, prospective studies should investigate whether PBBM–cerebral physiology relationships differ meaningfully across TBI phenotypes.

## Conclusion

PBBM levels were linked to key cerebral physiological disturbances during intensive care of TBI patients. Early PBBM concentrations were associated with the subsequent burden of ICP, PRx, and ΔCPPopt defined insults, while median PBBM levels over the first week also correlated with the cumulative load of such cerebral physiological insults. Temporal analyses indicated a stronger association when cerebral physiology was analyzed simultaneously with PBBM sampling. Elevated ICP showed the most consistent and robust associations across all evaluated PBBMs, whereas impaired CPA (high PRx) tended to amplify some of the PBBM response to increased ICP. No single PBBM clearly outperformed the others in reflecting cerebral physiological or CPA dysfunction, although NfL appeared less responsive, likely due to its distinct release and clearance kinetics. Overall, these findings highlight the potential for PBBMs to serve as surrogate short-term endpoints in future trials for CPA-targeted interventions, but these findings should not be regarded as conclusive, instead warrant further validation in larger, preferably multicenter cohorts with high frequency sampling before/after intervention and/or insults. Importantly, our results highlight the potential for PBBMs to complement multimodal neuromonitoring by tracking secondary injury trajectories.

## Supplementary Information

Below is the link to the electronic supplementary material.

Supplementary Material 1.

## Data Availability

No datasets were generated or analysed during the current study.

## Abbreviations

ABP: Arterial Blood Pressure
ASDH: Acute Subdural Hematoma
BH: Benjamini-Hochberg
BTF: Brain Trauma Foundation
CENTER-TBI: Collaborative European NeuroTrauma Effectiveness Research in TBI
CI: Confidence Interval
CNS: Central Nervous System
COGiTATE CPPOpt: Guided Therapy: Assessment of Target Effectiveness
CPA: Cerebral Pressure Autoregulation
CPP: Cerebral Perfusion Pressure
CPPopt: Optimal Cerebral Perfusion Pressure
ΔCPPopt: =Cerebral Perfusion Pressure – Optimal Cerebral Perfusion Pressure
CT: Computed Tomography
EDH: Epidural Hematoma
GCS: Glasgow Coma Scale
GCS M: Glasgow Coma Scale Motor
GFAP: Glial Fibrillary Acidic Protein
%GMT: Percentage Good Monitoring Time
GOSE: Glasgow Outcome Scale Extended
ICP: Intracranial Pressure
ICU: Intensive Care Unit
ISS: Injury Severity Score
IQR: Interquartile Range
IVH: Intraventricular Hemorrhage
LMEM: Linear Mixed Effects Models
LOESS: Locally Estimated Scatterplot Smoothing
MAP: Mean Arterial Pressure
NfL: Neurofilament Light Chain
NSE: Neuron-Specific Enolase
PBBM: Proteomic Blood-Based Biomarker
PRx: Pressure Reactivity Index
SE: Standard Error
S100B S100: Calcium-Binding Protein B
SiMoA: Single-Molecule Arrays
tSAH: Traumatic Subarachnoid Hemorrhage
t-Tau: Total Tubulin Associated Unit
TBI: Traumatic Brain Injury
UCH-L1: Ubiquitin C-terminal Hydrolase-L1
