# Supplementary material for: Proteomic blood-based biomarkers of brain damage in traumatic brain injury: are they suitable surrogate endpoints for cerebral pressure autoregulatory-guided therapy?
Source: Crit Care. 2026 Jun 13;30:311. doi: 10.1186/s13054-026-06132-4 (PMC13267589; doi:10.1186/s13054-026-06132-4)
Supplement: Supplementary file 1 — Supplementary Material 1. [file 13054_2026_6132_MOESM1_ESM.docx]

**Supplemental methods**

**Visualization methods**

Custom written R-scripts were used to visualize the presented results. First, to map potential correlations between PBBMs and cerebral physiological variables, a grid cell heatmap of correlations between GFAP, NfL, NSE, S100B, t-Tau, and UCH-L1 with ICP, PRx, CPP, and ΔCPPopt was created (Fig. 1). The median of each PBBM and cerebral physiological variable was calculated up to a maximum of seven days post-injury. Spearman’s correlation coefficient was calculated across all combinations. Each cell was color-coded from red (negative correlation) or blue (positive correlation), and the color-scale was set to -0.5 to 0.5 for visual representation. Statistically significant values (p < 0.05) were bolded, where BH correction was applied and reported as *q* values too. The same principle was applied for %GMT of PRx > 0.2, CPP < 60 mmHg, CPP 60-70 mmHg, CPP > 70 mmHg, ΔCPPopt < -5 mmHg, ΔCPPopt -5 to 5 mmHg, and ΔCPPopt > 5 mmHg and PBBM concentrations (Supplemental Fig. 3). To explore the association of PBBM values on the first day post-injury with the burden of cerebral physiological insults on the subsequent first week, day 1-values of PBBMs were correlated against day 1-7 values of %GMT for each cerebral physiological variable and visualized in the same way (Supplemental Fig. 4).

Second, to examine the temporal patterns, a cross correlation plot was constructed of Spearman’s correlation coefficient with 95% CI between PBBM concentrations and cerebral physiological variables with time lags of 24-hours, throughout day 1-7 after injury (Fig. 2). Cerebral physiology was measured centered around the PBBM collection ± 12 hours. To obtain one value, the median was calculated per patient, per day, and per lag. All PBBM values (per patient, per day) were matched with the corresponding physiological values (per patient, per day, and per lag). The lags represent a shift of cerebral physiological variables in relation to PBBM collection. Then, Spearman’s correlation coefficient and 95% CI were calculated for each PBBM-physiological variable pair across all lags. Due to limited data availability the lags -5 to 5 were used. Cross correlation plots were visualized using *ggplot2*. For each PBBM–physiological variable pair across the examined lags, the Spearman correlation coefficients were displayed in faceted scatter plots with error bars representing 95% CI. A horizontal and vertical dashed line were added, representing correlation coefficient 0 and lag 0 respectively. The same principle was applied with predefined time windows (0.5, 1, 2, and 6 hours) based on medians of physiological variables gathered both before and after PBBM collection (Supplemental Fig. 5).

Third, to adjust for repeated measures and further explore the association between PBBMs and cerebral physiological variables, we performed a series of univariate linear mixed effects model (LMEM) (Fig. 3). Each PBBM was separately modelled as the dependent variable, with each cerebral physiological variable included as a fixed effect. Analyses were restricted to day 1-7 of both PBBM and cerebral physiological data collection. A random intercept for each patient was included to adjust for repeated measures within individuals. For each model, scatterplots were generated showing the log10-transformed PBBM levels on the y-axis and the corresponding cerebral physiological variable on the x-axis, overlayed with the LMEM fit (dashed red line). Out of bonds data were visualized using the *oob squish* function for both axes. The data density was calculated and visualized using a color-scale from blue (low) to yellow (high), using the *geom_pointdensity* function in the R-package *ggplot2*. The color-scale was autofitted. The slope, standard error, and *p* value for the fixed effect was annotated in each plot. BH correction was applied and reported as *q* values. Models were fit using the *lmer* function, included in the *lme4* R-package, and *p* values were obtained using the *lmerTest* function.

Fourth, to highlight the data frequency during the first seven days of following injury, scatterplots were created of PBBM concentrations (blue) and values of cerebral physiological variables (red) (Supplemental Fig. 2). Each PBBM concentration was logged with base ten to improve visualization, as well as the daily median value for each physiological variable was used. *Oob squish* was utilized to handle outlier data. Lastly, local regression with LOESS was calculated and visualized (black line) to highlight the temporal data trend. The scatterplot was created using the R-package *ggplot2*, and ordered using the function *facet* *wrap*.

**Supplemental table 1. Proteomic blood-based biomarkers secondary peak vs. no secondary peak**

| Variable | GFAP | | | | NfL | | | | NSE | | | | S100B | | | | t-Tau | | | | UCH-L1 | | | |
| --- | --- | --- | --- | --- | --- | --- | --- | --- | --- | --- | --- | --- | --- | --- | --- | --- | --- | --- | --- | --- | --- | --- | --- | --- |
|  | No secondary  Peak (n=55) | Secondary  Peak (n=63) | *p* value | *q* value | No secondary  peak (n =11) | Secondary  peak (n=107) | *p* value | *q* value | No secondary  peak (n=34) | Secondary  peak (n=84) | *p* value | *q* value | No secondary  Peak  (n=71) | Secondary  Peak (n=47) | *p* value | *q* value | No secondary  peak (n=49) | Secondary  peak (n=69) | *p* value | *q* value | No secondary  peak (n=44) | Secondary  peak (n=74) | *p* value | *q* value |
| ICP (mmHg), median (IQR) | 11.20 (8.90-14.17) | 13.33 (10.79-15.93) | **0.016** | 0.208 | 12.38 (6.90-17.42) | 12.55 (9.15-15.43) | 0.740 | 0.740 | 12.55 (9.11-17.34) | 12.52 (9.33-15.21) | 0.893 | 0.893 | 12.52 (9.42-15.64) | 12.63 (8.48-15.43) | 0.808 | 0.956 | 11.20 (8.95-15.06) | 13.07 (10.63-15.81) | 0.083 | 0.252 | 11.27 (8.13-16.18) | 12.59 (10.63-14.92) | 0.374 | 0.486 |
| ICP > 22 mmHg (%GMT), median (IQR) | 2.67 (0.58-6.38) | 2.15 (0.46-9.68) | 0.690 | 0.928 | 6.73 (0.50-13.93) | 2.27 (0.52-7.11) | 0.422 | 0.610 | 3.81 (1.28-14.02) | 1.89 (0.40-6.41) | 0.070 | 0.725 | 2.81 (0.80-7.39) | 1.77 (0.40-8.18) | 0.635 | 0.956 | 2.67 (0.54-6.54) | 2.35 (0.52-8.52) | 0.650 | 0.785 | 3.43 (0.92-13.49) | 2.00 (0.41-6.73) | 0.235 | 0.382 |
| PRx, median (IQR) | -0.01 (-0.17-0.10) | 0.00 (-0.12-0.20) | 0.118 | 0.767 | 0.00 (-0.13-0.08) | 0.00 (-0.14-0.13) | 0.559 | 0.716 | -0.05 (-0.16-0.07) | 0.01 (-0.13-0.14) | 0.135 | 0.725 | -0.01 (-0.13-0.10) | 0.02 (-0.14-0.17) | 0.318 | 0.936 | 0.01 (-0.16-0.12) | -0.01 (-0.12-0.13) | 0.576 | 0.785 | 0.01 (-0.18-0.13) | 0.00 (-0.11-0.13) | 0.570 | 0.674 |
| PRx > 0.2 (%GMT), median (IQR) | 29.70 (18.85-39.30) | 28.02 (21.45-49.94) | 0.336 | 0.928 | 29.46 (19.76-36.53) | 28.53 (20.75-42.58) | 0.661 | 0.716 | 27.88 (19.76-37.68) | 29.46 (21.35-43.61) | 0.277 | 0.725 | 29.24 (20.16-39.68) | 29.53 (21.83-46.66) | 0.360 | 0.936 | 30.04 (19.68-41.88) | 27.52 (21.32-42.51) | 0.960 | 0.960 | 29.70 (18.73-41.88) | 28.24 (21.39-42.51) | 0.924 | 0.924 |
| CPP (mmHg), median (IQR) | 68.66 (62.63-76.79) | 68.00 (62.95-76.04) | 0.965 | 0.965 | 74.50 (67.05-77.54) | 68.06 (62.49-75.42) | 0.270 | 0.585 | 69.20 (62.47-76.28) | 68.13 (63.06-77.49) | 0.519 | 0.725 | 68.13 (62.94-76.62) | 70.81 (62.92-76.62) | 0.503 | 0.956 | 70.46 (65.70-78.04) | 67.07 (61.79-74.50) | **0.044** | 0.191 | 70.80 (64.38-79.48) | 67.43 (62.49-74.29) | 0.160 | 0.297 |
| CPP < 60 mmHg (%GMT), median (IQR) | 15.00 (4.02-38.21) | 15.60 (3.69-38.43) | 0.876 | 0.949 | 11.84 (2.75-25.79) | 16.30 (4.36-39.06) | 0.253 | 0.585 | 21.50 (5.61-39.10) | 13.91 (3.44-35.54) | 0.558 | 0.725 | 19.70 (5.58-38.93) | 11.96 (2.71-37.47) | 0.255 | 0.936 | 10.77 (2.74-29.42) | 24.35 (6.11-41.11) | **0.028** | 0.191 | 11.96 (2.29-31.79) | 23.86 (5.55-39.02) | 0.140 | 0.297 |
| CPP 60-70 mmHg (%GMT), median (IQR) | 28.87 (20.34-35.87) | 29.21 (17.09-35.55) | 0.776 | 0.928 | 18.34 (14.15-28.79) | 29.48 (20.17-35.87) | 0.156 | 0.585 | 28.55 (21.88-33.30) | 29.21 (16.54-39.75) | 0.501 | 0.725 | 29.05 (20.92-38.94) | 29.02 (13.78-34.87) | 0.321 | 0.936 | 27.73 (14.58-35.00) | 29.41 (19.99-35.88) | 0.415 | 0.771 | 27.03 (13.67-33.72) | 30.11 (20.71-38.94) | 0.071 | 0.268 |
| CPP > 70 mmHg (%GMT), median (IQR) | 45.75 (26.61-73.64) | 42.45 (23.14-69.33) | 0.785 | 0.928 | 58.35 (39.89-81.96) | 43.48 (23.08-69.54) | 0.191 | 0.585 | 47.90 (26.19-68.87) | 42.45 (23.40-69.75) | 0.876 | 0.893 | 41.68 (22.89-69.41) | 52.64 (24.57-73.14) | 0.289 | 0.936 | 51.77 (33.48-82.77) | 38.73 (21.28-69.16) | **0.033** | 0.191 | 52.57 (28.11-84.04) | 39.76 (23.02-69.23) | 0.103 | 0.268 |
| CPPopt (mmHg), median (IQR) | 69.90 (64.68-78.47) | 70.90 (63.95-75.44) | 0.741 | 0.928 | 75.07 (68.28-82.94) | 69.97 (63.91-75.69) | 0.191 | 0.585 | 70.56 (64.33-78.47) | 69.67 (64.06-75.89) | 0.810 | 0.893 | 69.67 (64.46-75.89) | 72.14 (63.91-77.68) | 0.682 | 0.956 | 71.33 (67.70-79.01) | 69.45 (62.62-75.59) | 0.097 | 0.252 | 73.44 (67.04-80.54) | 69.46 (62.65-75.07) | **0.026** | 0.268 |
| ΔCPPopt (mmHg), median (IQR) | -0.05 (-2.39-1.51) | -0.29 (-0.308-1.56) | 0.706 | 0.928 | 1.28 (-3.76-2.58) | -0.18 (-3.00-1.40) | 0.634 | 0.716 | -0.73 (-2.81-1.07) | 0.08 (-3.02-1.95) | 0.264 | 0.725 | -0.15 (-3.30-1.80) | -0.17 (-2.33-1.29) | 0.956 | 0.956 | -0.05 (-3.04-1.59) | -0.57 (-3.02-1.46) | 0.489 | 0.785 | -0.73 (-3.85-1.71) | -0.35 (-2.17-1.46) | 0.282 | 0.407 |
| ΔCPPopt < -5 mmHg (%GMT), median (IQR) | 29.52 (23.71-40.89) | 31.68 (21.79-41.83) | 0.720 | 0.928 | 32.85 (26.35-44.14) | 31.04 (21.79-40.74) | 0.418 | 0.610 | 31.59 (27.68-42.87) | 30.27 (21.66-40.93) | 0.351 | 0.725 | 30.45 (21.57-42.64) | 31.49 (23.97-39.57) | 0.802 | 0.956 | 30.10 (23.80-41.82) | 31.83 (21.75-40.86) | 0.816 | 0.884 | 32.03 (27.37-44.41) | 29.51 (21.21-39.93) | 0.091 | 0.268 |
| ΔCPPopt ± 5 mmHg (%GMT), median (IQR) | 37.56 (32.84-43.21) | 38.00 (32.08-46.47) | 0.577 | 0.928 | 34.61 (32.07-35.04) | 38.16 (32.46-46.27) | 0.062 | 0.585 | 37.94 (34.61-45.80) | 37.42 (31.36-45.65) | 0.409 | 0.725 | 37.71 (32.84-46.00) | 36.51 (31.46-45.64) | 0.815 | 0.956 | 35.51 (32.91-43.85) | 39.93 (32.01-46.79) | 0.402 | 0.771 | 36.56 (31.56-41.19) | 39.95 (32.81-47.52) | 0.090 | 0.268 |
| ΔCPPopt > 5 mmHg (%GMT), median (IQR) | 27.56 (20.90-35.88) | 27.66 (16.22-36.65) | 0.728 | 0.928 | 35.89 (19.55-40.76) | 27.57 (18.70-35.31) | 0.397 | 0.610 | 27.09 (19.60-32.09) | 28.22 (17.62-37.46) | 0.422 | 0.725 | 27.13 (19.63-35.88) | 28.43 (15.54-36.64) | 0.889 | 0.956 | 27.56 (20.20-35.88) | 27.66 (17.92-36.94) | 0.664 | 0.785 | 25.15 (18.18-37.82) | 28.49 (20.15-35.86) | 0.798 | 0.864 |

GFAP = Glial Fibrillary Acidic Protein. NfL = Neurofilament Light Chain. NSE = Neuron-Specific Enolase. S100B = S100 calcium-binding protein B. t-Tau = total Tubulin associated unit. UCH-L1 = Ubiquitin C-terminal Hydrolase-L1. CPP = Cerebral Perfusion Pressure. CPPopt = Optimal CPP. ΔCPPopt = CPP – optimal CPP. GMT = Good Monitoring Time. ICP = Intracranial Cerebral Pressure. PRx = Pressure Reactivity Index. IQR = Interquartile range.

**Supplemental table 2. Proteomic blood-based biomarkers vs. Cerebral physiological variables during the first seven days post-injury – A multivariate linear mixed effects model analysis (hypo- vs hyperperfusion)**

| Variable | GFAP | | | NfL | | | NSE | | | S100B | | | t-Tau | | | UCH-L1 | | |
| --- | --- | --- | --- | --- | --- | --- | --- | --- | --- | --- | --- | --- | --- | --- | --- | --- | --- | --- |
|  | Value ± SE | *p* value | *q* value | Value ± SE | *p* value | *q* value | Value ± SE | *p* value | *q* value | Value ± SE | *p* value | *q* value | Value ± SE | *p* value | *q* value | Value ± SE | *p* value | *q* value |
| ICP | 0.022 ± 0.004 | **<0.001** | **<0.001** | 0.010 ± 0.003 | **0.001** | **0.008** | 0.006 ± 0.002 | **0.003** | **0.014** | 0.027 ± 0.005 | **<0.001** | **<0.001** | 0.012 ± 0.004 | **0.002** | **0.011** | 0.014 ± 0.005 | **0.003** | **0.013** |
| PRx | 0.070 ± 0.141 | 0.620 | 0.761 | -0.319 ± 0.095 | <**0.001** | **0.007** | 0.066 ± 0.069 | 0.343 | 0.562 | 0.405 ± 0.172 | **0.019** | 0.061 | 0.090 ± 0.134 | 0.502 | 0.695 | 0.126 ± 0.150 | 0.401 | 0.620 |
| ΔCPPopt_negative_ | -0.005 ± 0.004 | 0.302 | 0.543 | 0.000 ± 0.003 | 0.894 | 0.947 | 0.006 ± 0.003 | **0.029** | 0.087 | 0.011 ± 0.007 | 0.086 | 0.221 | 0.000 ± 0.005 | 0.923 | 0.958 | 0.001 ± 0.005 | 0.804 | 0.893 |
| ΔCPPopt_positive_ | -0.004 ± 0.005 | 0.422 | 0.626 | 0.002 ± 0.004 | 0.659 | 0.791 | -0.002 ± 0.003 | 0.620 | 0.761 | -0.005 ± 0.008 | 0.535 | 0.722 | -0.003 ± 0.006 | 0.556 | 0.732 | -0.001 ± 0.006 | 0.810 | 0.893 |
| ICP:PRx | -0.012 ± 0.009 | 0.199 | 0.429 | 0.020 ± 0.006 | **0.001** | **0.008** | 0.010 ± 0.004 | **0.016** | 0.054 | 0.012 ± 0.011 | 0.284 | 0.528 | 0.010 ± 0.009 | 0.267 | 0.517 | -0.001 ± 0.009 | 0.878 | 0.947 |
| PRx:ΔCPPopt_negative_ | 0.024 ± 0.013 | 0.062 | 0.167 | 0.009 ± 0.010 | 0.327 | 0.562 | -0.005 ± 0.007 | 0.440 | 0.626 | -0.068 ± 0.017 | **<0.001** | **0.002** | 0.011 ± 0.013 | 0.402 | 0.620 | 0.016 ± 0.015 | 0.268 | 0.517 |
| PRx:ΔCPPopt_positive_ | 0.000 ± 0.020 | 0.983 | 0.998 | -0.009 ± 0.010 | 0.570 | 0.733 | -0.004 ± 0.013 | 0.743 | 0.853 | -0.037 ± 0.031 | 0.230 | 0.477 | -0.016 ± 0.020 | 0.429 | 0.626 | 0.000 ± 0.023 | 0.998 | 0.998 |
| Age | 0.009 ± 0.002 | **<0.001** | **0.002** | -0.001 ± 0.002 | 0.675 | 0.792 | -0.002 ± 0.001 | **0.045** | 0.128 | 0.008 ± 0.002 | **0.008** | **0.011** | 0.004 ± 0.002 | **0.015** | 0.052 | 0.005 ± 0.002 | **0.010** | **0.040** |
| GCS M at admission | -0.034 ± 0.025 | 0.174 | 0.408 | -0.049 ± 0.016 | **0.003** | **0.014** | -0.030 ± 0.008 | **<0.001** | **0.005** | -0.023 ± 0.024 | 0.341 | 0.562 | -0.022 ± 0.017 | 0.182 | 0.410 | -0.031 ± 0.020 | 0.126 | 0.308 |

GFAP = Glial Fibrillary Acidic Protein. NfL = Neurofilament Light Chain. NSE = Neuron-Specific Enolase. S100B = S100 calcium-binding protein B. t-Tau = total Tubulin associated unit. UCH-L1 = Ubiquitin C-terminal Hydrolase-L1. CPP = Cerebral Perfusion Pressure. CPPopt = Optimal CPP. ΔCPPopt_positive_ = CPP – optimal CPP. ΔCPPopt_negative_ = |CPP – optimal CPP|. GMT = Good Monitoring Time. ICP = Intracranial Cerebral Pressure. PRx = Pressure Reactivity Index. IQR = Interquartile range.

**Supplemental fig 1. Flowchart of patient inclusion**


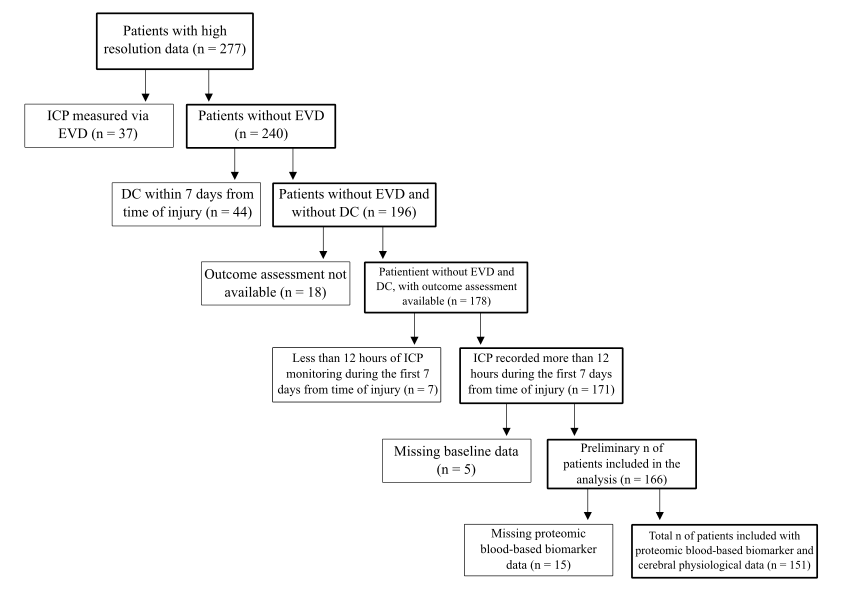


The flowchart illustrates the patient inclusion of this study. Initially, 277 patients with high-frequency data were evaluated for eligibility. The evaluation resulted in exclusion of 111 patients; 37 patients with ICP recorded via an external ventricular drain (EVD); 44 patients who underwent decompressive craniectomy (DC) within seven days from time of injury; 18 patients with no Extended Glasgow Outcome Scale (GOSE) assessment at six months post-injury; 7 patients with less than 12 hours of ICP monitoring during the first seven days from time of injury, and 5 patients with missing baseline clinical variables. Of the 166 patients left, 15 patients had unavailable PBBMs of GFAP, NfL, NSE, S100B, t-Tau, and UCH-L1, at admission and over the NIC course. Hence, 151 TBI patients were eligible for inclusion in this study.

**Supplemental fig 2. Temporal dynamics during the first seven days post-injury**

**2a. Proteomic blood-based biomarkers**


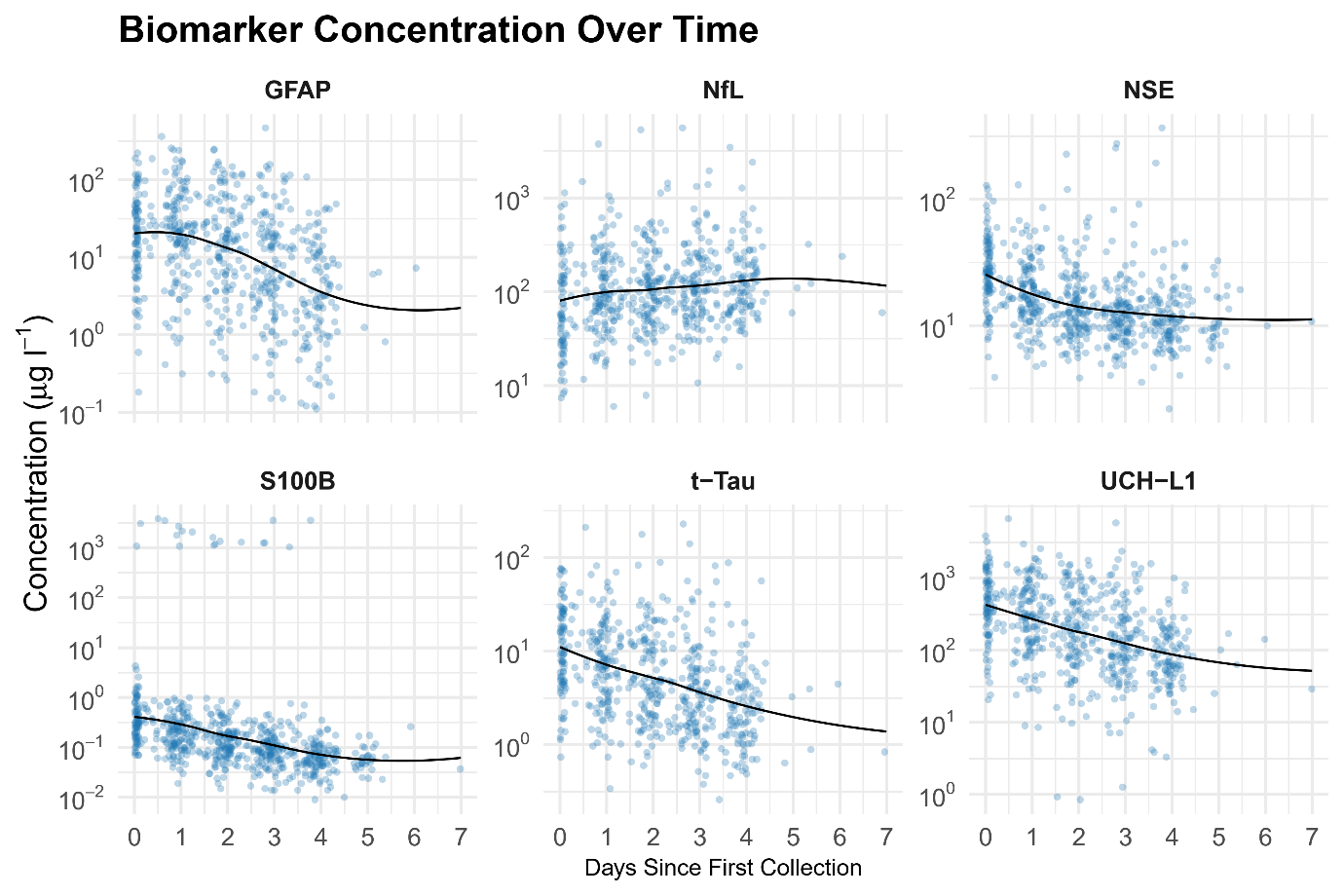


**2b. Cerebral physiological variables**


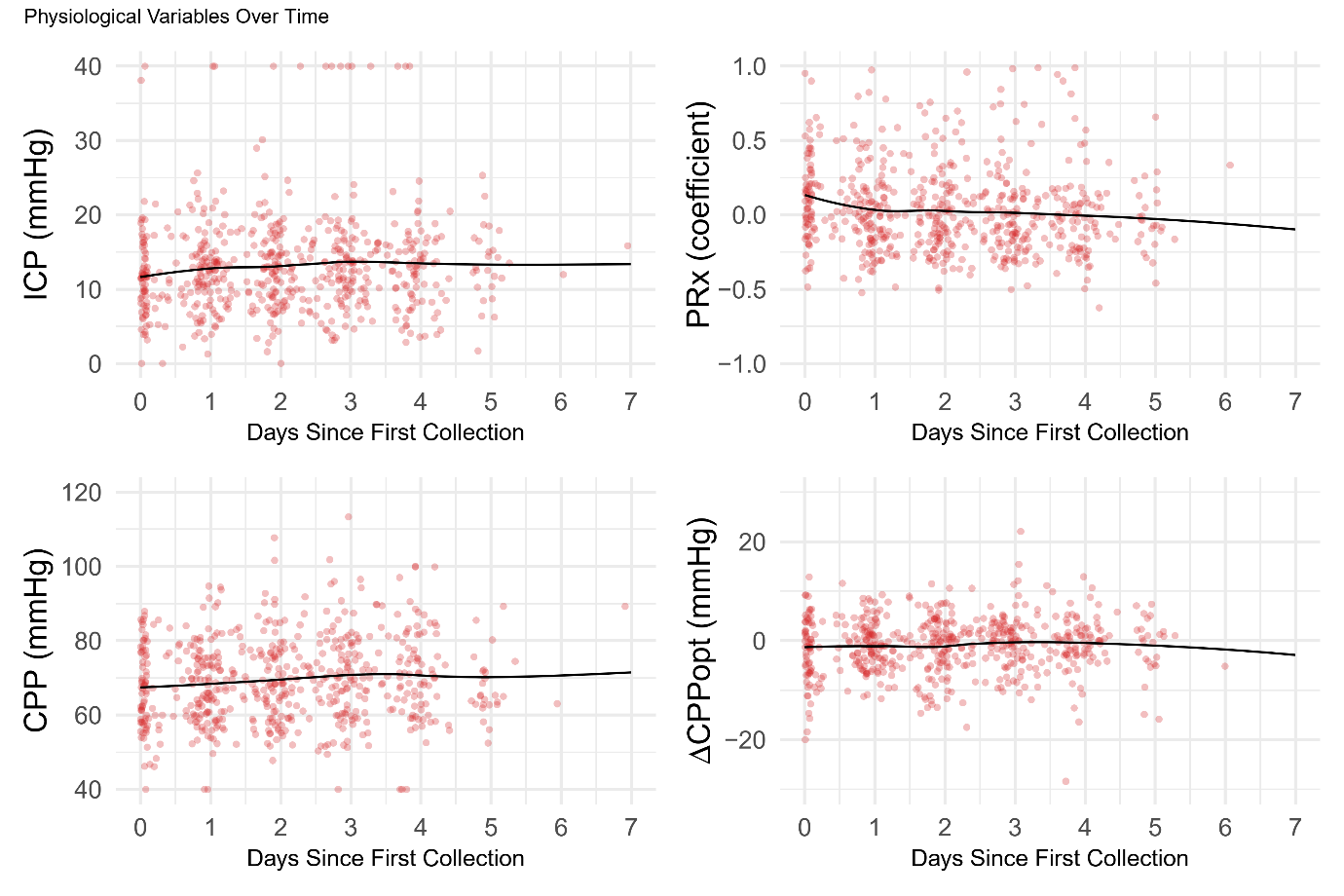


Scatterplots demonstrating the concentration of GFAP, NfL, NSE, S100B, t-Tau, and UCH-L1 (2a) as well as data for ICP, PRx, CPP, and ΔCPPopt (2b) during the first seven days of intensive care following injury. The black line is based on local regression with LOESS and highlights the temporal change in trend. For all cerebral physiological variables, the trend was neutral, meanwhile a downwards trend was visible for all PBBMs except for NfL, which demonstrated an upwards trend instead.

**Supplemental fig 3. The association between proteomic blood-based biomarkers and %GMT of cerebral physiological variables – A Spearman correlation matrix of data gathered the first seven days post-injury**


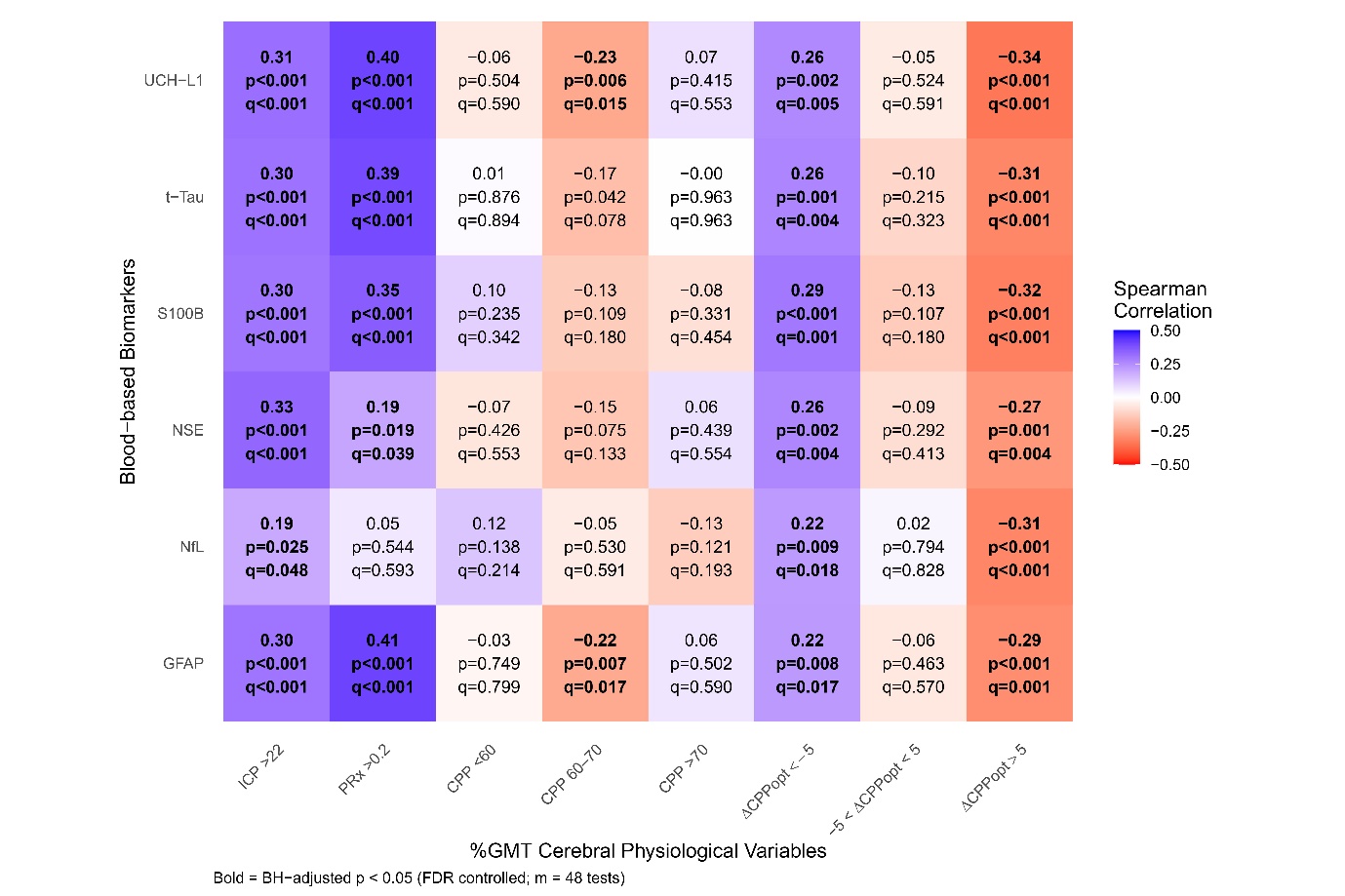


Correlation matrix between PBBMs (GFAP, NfL, NSE, S100B, t-Tau, and UCH-L1) and %GMT of cerebral physiological variables (ICP above 22 mmHg, PRx above 0.2, CPP below 60 mmHg, CPP between 60 to 70 mmHg, CPP above 70 mmHg, ΔCPPopt below -5 mmHg, ΔCPPopt between -5 to 5 mmHg, ΔCPPopt above 5 mmHg) using Spearman correlation analysis. To illustrate the correlation coefficient, grid cells were color-coded from red (negative) to blue (positive). Statistically significant values (*p* and *q* < 0.05) are bolded. A positive and statistically significant correlation was seen for GFAP and ICP > 22 mmHg, PRx > 0.2, and ΔCPPopt < -5 mmHg; for NfL and ICP > 22 mmHg, ΔCPPopt < -5 mmHg; for NSE and ICP > 22 mmHg, PRx > 0.2 and ΔCPPopt < -5 mmHg; for S100B and ICP > 22 mmHg, PRx > 0.2, and ΔCPPopt < -5 mmHg; for t-Tau and ICP > 22 mmHg, PRx > 0.2, and ΔCPPopt < -5 mmHg; for UCH-L1 and ICP > 22 mmHg, PRx > 0.2, and ΔCPPopt < -5 mmHg. A negative correlation was seen for GFAP and CPP 60-70 mmHg, ΔCPPopt > 5 mmHg; for NfL and ΔCPPopt > 5 mmHg; for NSE and ΔCPPopt > 5 mmHg; for S100B and ΔCPPopt > 5 mmHg; for t-Tau and ΔCPPopt > 5 mmHg; for UCH-L1 and CPP 60-70 mmHg, ΔCPPopt > 5 mmHg.

**Supplemental fig 4. The association between day 1 proteomic blood-based biomarkers and day 1-7 %GMT of cerebral physiological variables – A Spearman correlation matrix**


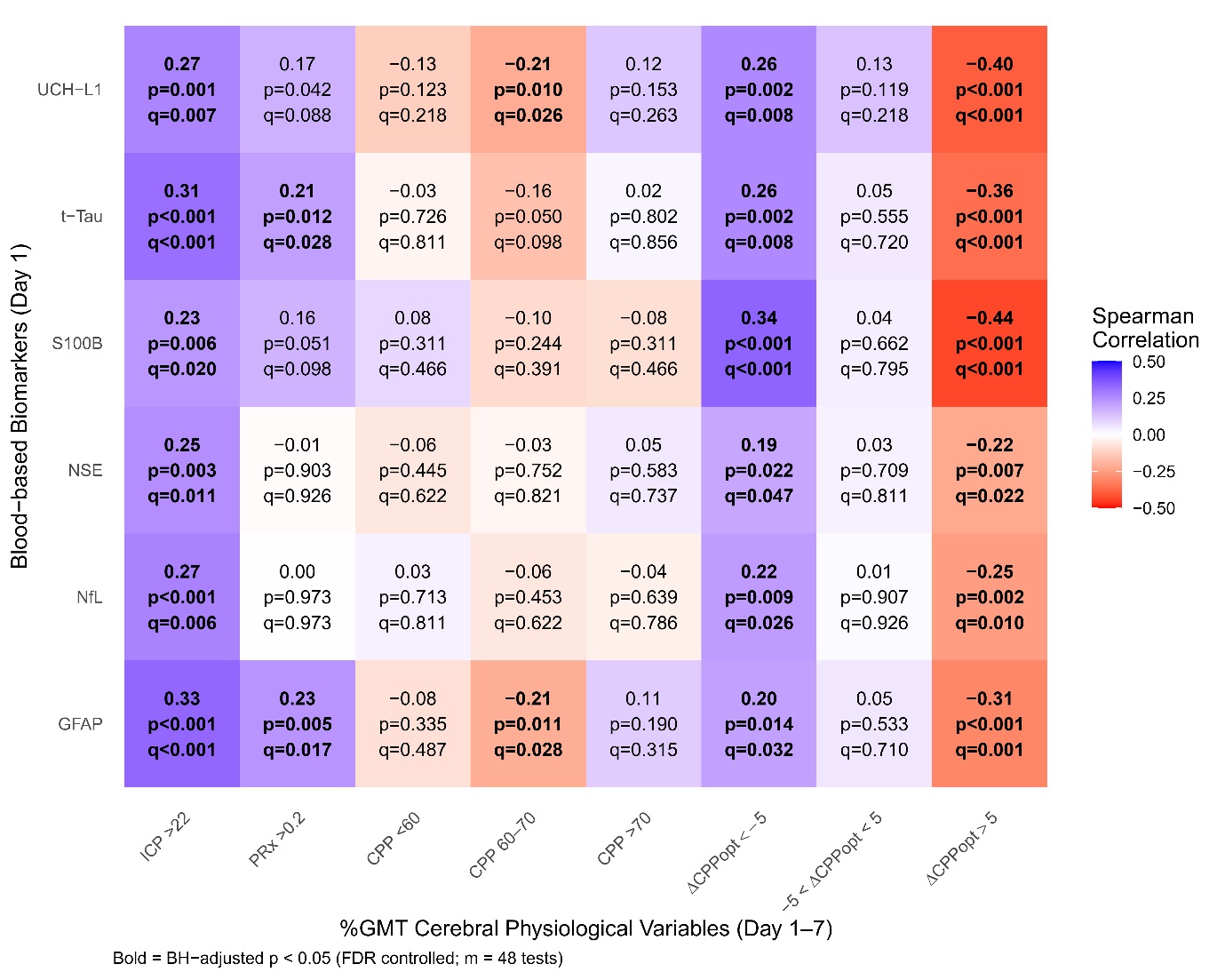


Correlation matrix between day 1 values of PBBMs (GFAP, NfL, NSE, S100B, t-Tau, and UCH-L1) and %GMT of cerebral physiological variables day 1-7 (ICP > 22 mmHg, PRx > 0.2, CPP < 60 mmHg, CPP 60-70 mmHg, CPP > 70 mmHg, ΔCPPopt < -5 mmHg, ΔCPPopt -5 to 5 mmHg, ΔCPPopt > 5 mmHg) using Spearman correlation analysis. To illustrate the correlation coefficient, grid cells were color-coded from red (negative) to blue (positive). Statistically significant values (*p* and *q* < 0.05) are bolded. A positive and statistically significant correlation was seen for GFAP and ICP > 22 mmHg, PRx > 0.2, and ΔCPPopt < -5 mmHg; for NfL and ICP > 22 mmHg, ΔCPPopt < -5 mmHg; for NSE and ICP > 22 mmHg, ΔCPPopt <-5 mmHg; for S100B and ICP > 22 mmHg, ΔCPPopt <-5 mmHg; for t-Tau and ICP > 22 mmHg, PRx > 0.2, and ΔCPPopt < -5 mmHg; for UCH-L1 and ICP > 22 mmHg, ΔCPPopt < -5 mmHg. A negative correlation was seen for GFAP and CPP 60-70 mmHg, ΔCPPopt > 5 mmHg; for NfL and ΔCPPopt > 5 mmHg; for NSE and ΔCPPopt > 5 mmHg; for S100B and ΔCPPopt > 5 mmHg; for t-Tau and ΔCPPopt > 5 mmHg; for UCH-L1 and CPP 60-70 mmHg, ΔCPPopt > 5 mmHg.

**Supplemental fig 5. Proteomic blood-based biomarkers vs. Cerebral physiological variables – Time windowed correlation**

**5a. Proteomic blood-based biomarkers vs. ICP**


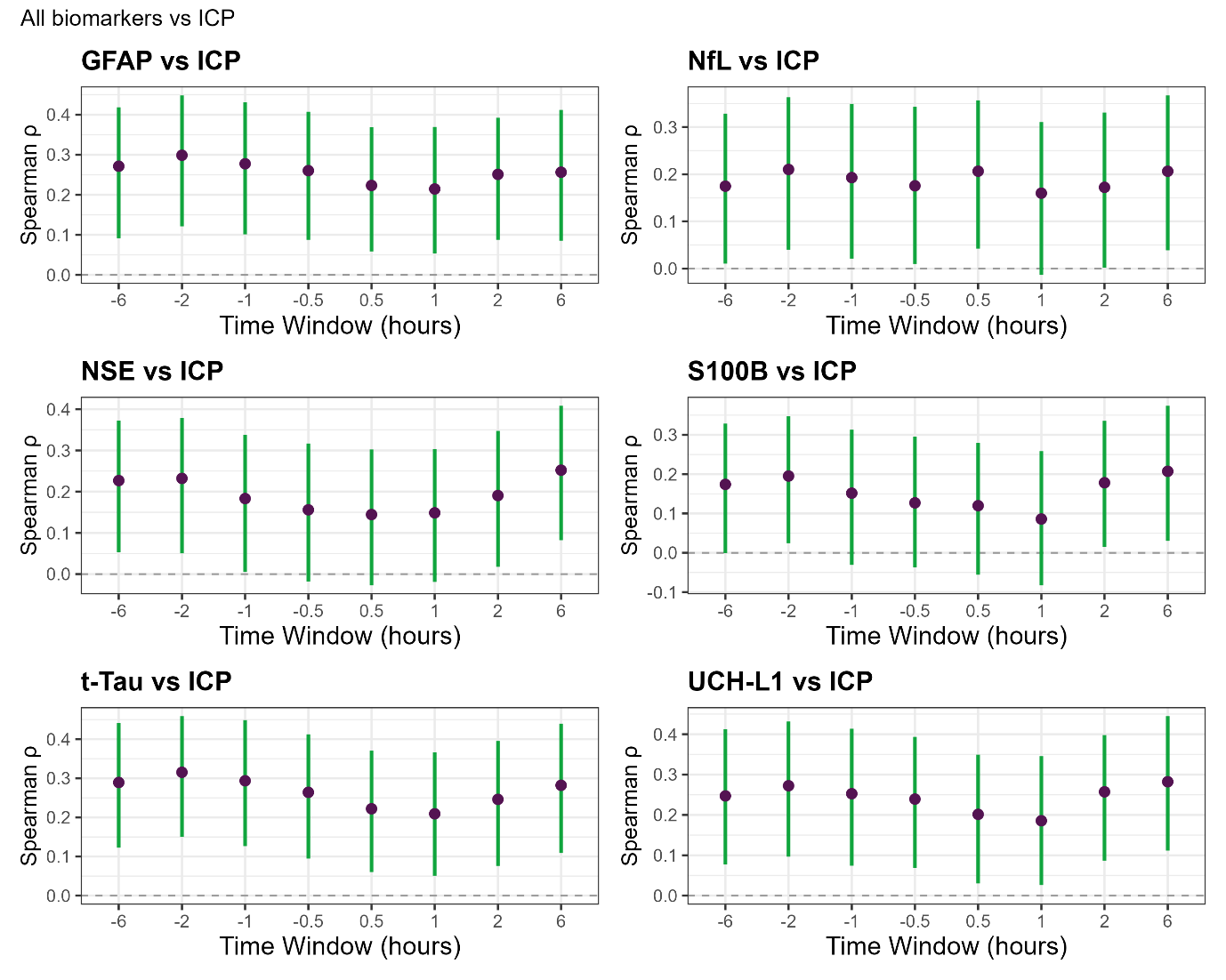


**5b. Proteomic blood-based biomarkers vs. PRx**
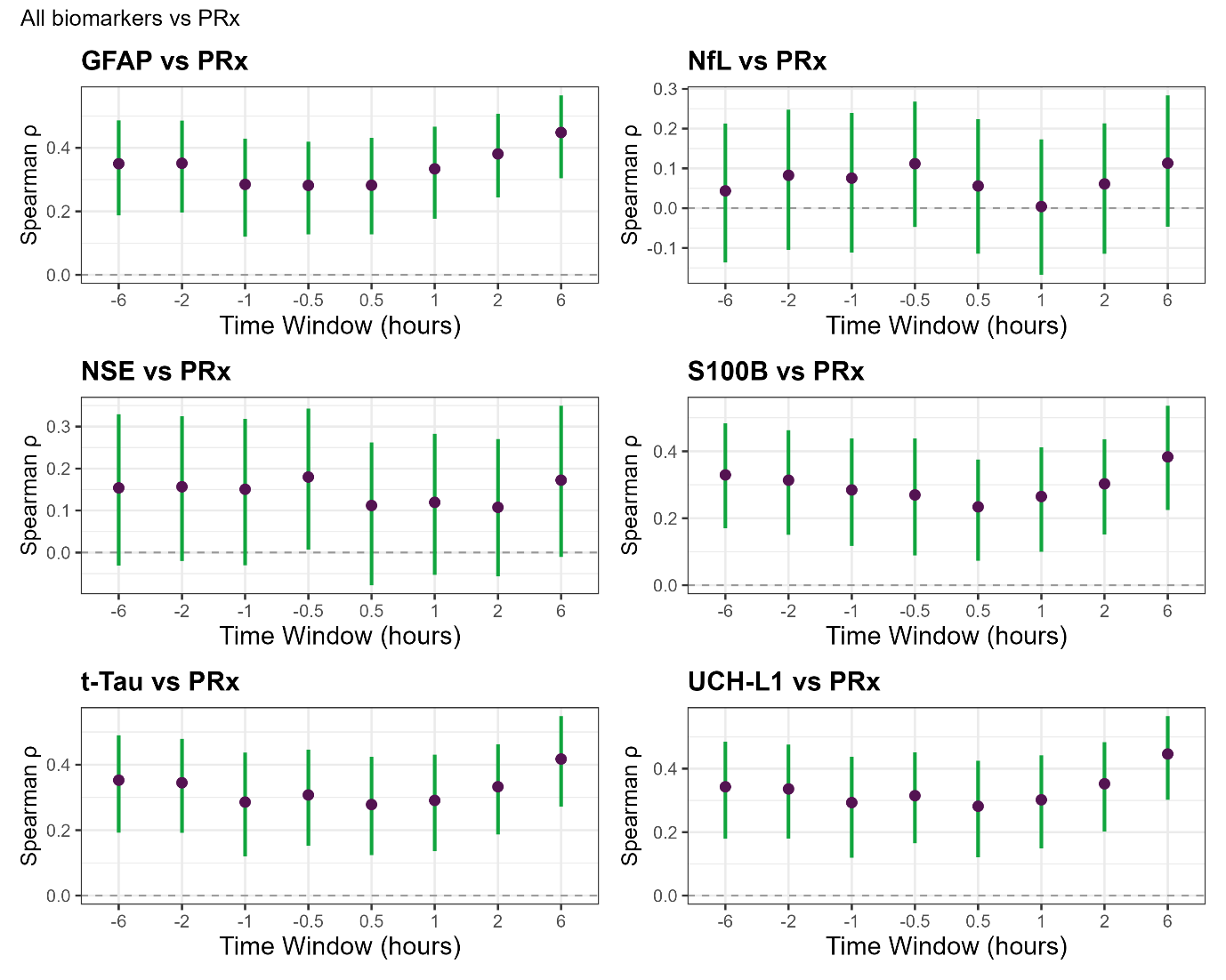


**5c. Proteomic blood-based biomarkers vs. CPP**


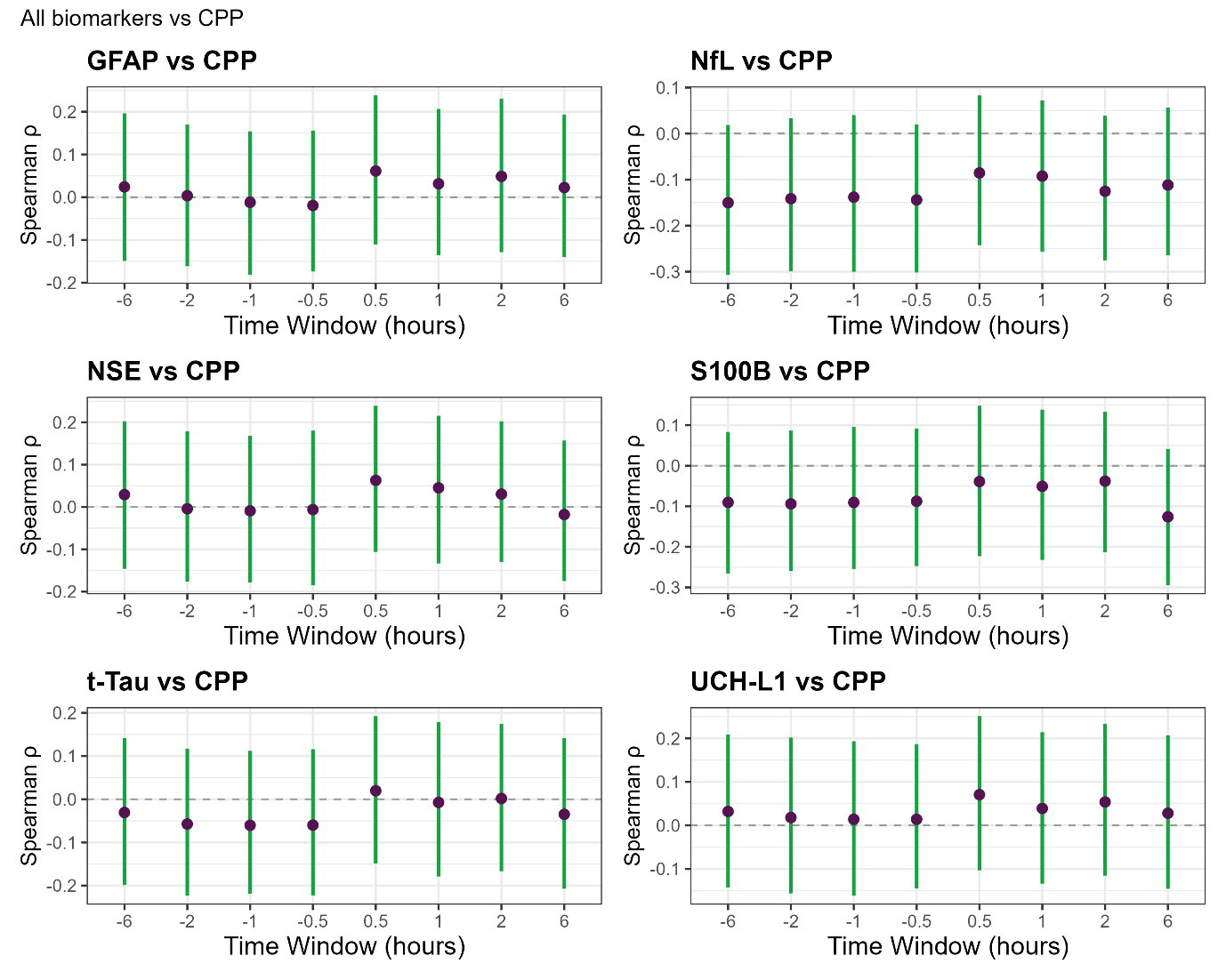


**5d. Proteomic blood-based biomarkers vs.** Δ**CPPopt**


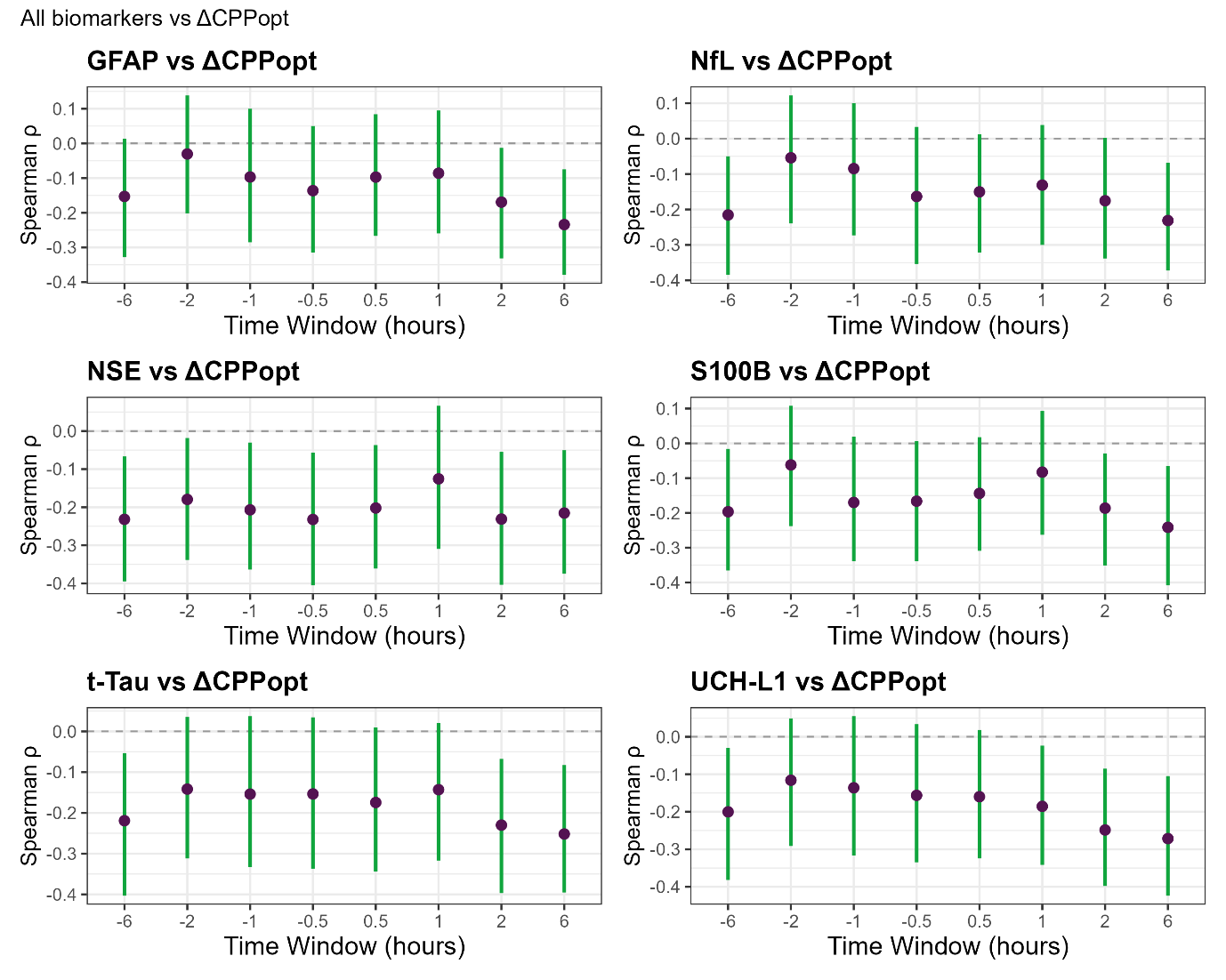


Cross correlation plot demonstrating the Spearman correlation coefficient with 95% CI between PBBM concentration (GFAP, NfL, NSE, S100B, t-Tau, and UCH-L1) and cerebral physiological variables (ICP, PRx, CPP, and ΔCPPopt) gathered at 0.5, 1, 2, and 6 hours before/after PBBM collection. As visualized, ICP showed only positive correlation for GFAP, t-Tau, and UCH-L1 regardless of time window, except 1 hour after for NfL, 0.5 hours before and 0.5 or 1 hour after for NSE, and 0.5 or 1 hour before and after for S100B. PRx demonstrated positive correlations for all time windows when correlated with GFAP, S100B, t-Tau, and UCH-L1. For CPP no clear strictly positive or negative correlation was demonstrated. ΔCPPopt displayed mainly negative correlations with all PBBMs.
